# *In vitro* analyses of mitochondrial ATP/phosphate carriers from *Arabidopsis thaliana* revealed unexpected Ca^2+^-effects

**DOI:** 10.1186/s12870-015-0616-0

**Published:** 2015-10-06

**Authors:** André Lorenz, Melanie Lorenz, Ute C. Vothknecht, Sandra Niopek-Witz, H. Ekkehard Neuhaus, Ilka Haferkamp

**Affiliations:** Cellular Physiology/Membrane Transport, University of Kaiserslautern, 67653 Kaiserslautern, Germany; Department of Biology I, Botany, LMU Munich, Großhaderner Str. 2, D-82152 Planegg-Martinsried, Germany; Plant Physiology, University of Kaiserslautern, 67653 Kaiserslautern, Germany

**Keywords:** Mitochondria, calcium, Ca^2+^, Signaling, Energy, Adenine nucleotide transport, Plant, ATP, ADP, Phosphate

## Abstract

**Background:**

Adenine nucleotide/phosphate carriers (APCs) from mammals and yeast are commonly known to adapt the mitochondrial adenine nucleotide pool in accordance to cellular demands. They catalyze adenine nucleotide - particularly ATP-Mg - and phosphate exchange and their activity is regulated by calcium. Our current knowledge about corresponding proteins from plants is comparably limited. Recently, the three putative APCs from *Arabidopsis thaliana* were shown to restore the specific growth phenotype of APC yeast loss-of-function mutants and to interact with calcium via their N-terminal EF-hand motifs *in vitro*. In this study, we performed biochemical characterization of all three APC isoforms from *A. thaliana* to gain further insights into their functional properties*.*

**Results:**

Recombinant plant APCs were functionally reconstituted into liposomes and their biochemical characteristics were determined by transport measurements using radiolabeled substrates. All three plant APCs were capable of ATP, ADP and phosphate exchange, however, high preference for ATP-Mg, as shown for orthologous carriers, was not detectable. By contrast, the obtained data suggest that in the liposomal system the plant APCs rather favor ATP-Ca as substrate. Moreover, investigation of a representative mutant APC protein revealed that the observed calcium effects on ATP transport did not primarily/essentially involve Ca^2+^-binding to the EF-hand motifs in the N-terminal domain of the carrier.

**Conclusion:**

Biochemical characteristics suggest that plant APCs can mediate net transport of adenine nucleotides and hence, like their pendants from animals and yeast, might be involved in the alteration of the mitochondrial adenine nucleotide pool. Although, ATP-Ca was identified as an apparent import substrate of plant APCs *in vitro* it is arguable whether ATP-Ca formation and thus the corresponding transport can take place *in vivo*.

**Electronic supplementary material:**

The online version of this article (doi:10.1186/s12870-015-0616-0) contains supplementary material, which is available to authorized users.

## Background

The mitochondrial carrier family (MCF) comprises structurally related but functionally diverse proteins that are characteristic for and generally restricted to eukaryotes [1–5]. MCF proteins represent the main solute carriers in the inner mitochondrial membrane and catalyze the translocation of various metabolites, such as nucleotides, cofactors, carboxylates, amino acids etc (for review see [6]).

Mitochondrial ATP-Mg/phosphate carriers (APCs) represent a specific MCF subgroup comprising carriers from different eukaryotes that are phylogenetically related to the well characterized ADP/ATP carriers (AACs) required for mitochondrial energy passage (for review see [6, 7]). Over the past years the physiological and biochemical properties of the single yeast APC isoform Sal1p (suppressor of ∆*aac2* lethality) as well as of various mammalian homologs became more and more clarified [8]. Initially, Sal1p was shown to suppress the growth phenotype of yeast impaired in mitochondrial energy transport (due to AAC deletion or inhibition). In a similar fashion, AAC compensates the loss of functional Sal1p [8]. Subsequent studies revealed that Sal1p and its mammalian homologs mediate the counter exchange of adenine nucleotides and phosphate [9–13]. Therefore, the redundant physiological function of Sal1p and AAC supposedly was not primarily energy exchange but adenine nucleotide translocation, most likely ATP entry into mitochondria [13, 14].

Alteration of the mitochondrial adenine nucleotide pool by adenine nucleotide exchange with phosphate was shown to affect different physiological processes, such as glucose metabolism, oxidative phosphorylation, mitochondrial biogenesis and DNA maintenance in yeast or mammals [9–13]. APC proteins apparently prefer two-fold negatively charged substrates, either ATP in complex with Mg^2+^ (ATP-Mg^2−^), protonated ADP (HADP^2−^) or HPO_4_^2−^, which makes the catalyzed transport electroneutral [15]. The composition (respective concentrations) of the different substrates at the matrix and cytosolic sides of the carrier determine whether adenine nucleotides preferentially become exported or imported [11, 15].

Interestingly, addition of Ca^2+^ to isolated mitochondria as well as metabolic situations that result in increase of free cytosolic Ca^2+^ were shown to enhance mitochondrial adenine nucleotide levels by stimulation of APC activity in mammals and yeast [8, 16–18] (for review see [19]). In one aspect APC proteins considerably differ structurally from typical MCF proteins; they are N-terminally extended by a domain that is exposed to the inter-membrane space of the mitochondrion and contains up to four putative Ca^2+^-binding EF-hand motifs [20–22]. Very recent structural studies with the N-terminal domain of human APC isoform 1 (also termed SCaMC1 for short Ca^2+^-dependent mitochondrial Carrier 1) showed that the Ca^2+^-bound state is quite compact and rigid whereas the apo (Ca^2+^-free) state appeared more flexible [21, 22]. Moreover, interaction studies with the two individual SCaMC1 domains, the Ca^2+^-binding part and the C-terminal transmembrane region, led to the assumption that the apo state of the N-terminal domain forms a cap that closes the translocation pathway whereas Ca^2+^-binding causes cap removal/opening and thus transporter activation [21, 22].

In contrast to yeast and mammals [8, 12, 16, 18, 23–25] analyses concerning the net adenine nucleotide transport of mitochondria in plants are still rudimentary. Previous studies led to controversial results but have indicated that plant mitochondria are capable of net adenine nucleotide uptake [26–31]. *Arabidopsis thaliana* possesses three putative APC proteins (*At*APC1-3) that exhibit high amino acid sequence similarities to their human and yeast counterparts. Phylogenetic analysis of MCF proteins shows that APCs cluster together and that plant APCs form a sister group to the human and yeast orthologs [6]. Similar to yeast or mammalian APCs, the plant pendants contain an N-terminal extension with four putative EF-hand motifs and were recently shown to interact with Ca^2+^ at least *in vitro* [32]. Moreover, all three plant isoforms were able to rescue the specific growth phenotype of ∆*sal1p* yeast mutants [32]. Therefore, *At*APC1-3 isoforms were suggested to represent Ca^2+^-regulated ATP-Mg/phosphate transporters. To gain first insights into the biochemical characteristics of the three APCs from *A. thaliana* we reconstituted the heterologously expressed proteins into liposomes and investigated their capacity for adenine nucleotide transport. Our data indicate that plant APCs mediate antiport of ATP, ADP and phosphate and therefore might be involved the alteration of the mitochondrial adenine nucleotide pool. Moreover, the determined transport characteristics suggest that in the *in vitro* system, the plant APCs preferentially import the Ca^2+^- and not the Mg^2+^-complexed form of ATP.

## Methods

### Generation of expression constructs

The coding sequences of *At*APC1-3 were amplified from *Arabidopsis* cDNA with specific primers via *Pfu*-polymerase-mediated PCR. For generation of the truncated *At*APC2 mutant protein lacking its Ca^2+^-interacting N-terminus a sense primer was chosen that internally hybridizes with the corresponding full-length sequence resulting in a recombinant protein starting at amino acid position 164 directly after the fourth predicted EF-hand motif coding region. The isopropyl β-D-thiogalactopyranoside (IPTG)-inducible T7 RNA polymerase pET-vector/Rosetta™ 2 expression system (Merck Biosciences, Novagen®, Darmstadt, Germany) was used for heterologous protein synthesis. Accordingly, the primers were adapted to allow insertion into the expression vector pET16b in frame with the histidine-tag coding sequence. The coding sequence of *At*APC1 was inserted via NdeI (sense primer) and XhoI (antisense primer) whereas the remaining sequences were inserted via XhoI (sense primer) and BamHI (antisense primer). Correctness of the respective expression constructs was verified by sequencing.

### Heterologous protein synthesis and detection

For heterologous protein synthesis Rosetta™ 2 cells were transformed with the expression constructs and cultured in 50 mL standard Terrific Broth (TB) medium at 37 °C under vigorous shaking. At an OD_600_ of 0.5, expression was induced by addition of 1 mM IPTG. Two hours after induction, cells were concentrated by centrifugation (3000 g, 5 min, 4 °C) and rapidly frozen (in liquid nitrogen). The frozen cell pellet was resuspended in buffer R (25 % sucrose, 50 mM Tris, pH 7.0, 1.5 % Triton X-100, 18.75 mM EDTA) supplemented with 1 mM PMSF, a pinch of DNAse and RNAse and incubated for approximately 30 min at 37 °C to stimulate autolysis by the endogenous lysozyme which was released from the cells due to the freeze/thaw procedure. Subsequent sonication additionally supported cell disruption. Inclusion bodies were separated from soluble and membrane proteins of the cell homogenate by centrifugation (20,000 *g*, 15 min, 4 °C).

For documentation of heterologous protein synthesis, an aliquot of the inclusion bodies fraction was used for SDS-PAGE, Western-blotting and immune detection. For this, inclusion bodies were resuspended in buffer R and an appropriate volume of 6 x concentrated sample buffer medium (375 mM Tris/HCl, pH 6.8, 0.3 % SDS, 60 % glycerol, 1.5 % bromophenol blue) was added. Protein separation was performed in a discontinuous, denaturing system with a 3 % stacking and a 12 % separating polyacrylamide gel [33]. Following electrophoresis, the gel was coomassie stained or used for Western-blotting. Immune detection was performed using a monoclonal anti poly His IgG (Sigma; http://www.sigmaaldrich.com) combined with a secondary alkaline phosphatase conjugated anti-mouse IgG (Sigma). Alkaline phosphatase activity was detected by staining with nitro blue tetrazolium chloride/5-bromo-4-chloro-3’-indoly phosphate toluidine salt.

### Purification of inclusion bodies

Basically, purification of inclusion bodies as well as their solubiliztaion, refolding and integration into lipid/detergent micelles was performed according to [34]. For this, the cell pellet of the inclusion body fraction washed in buffer W1 (20 ml 1 M urea, 1 % Triton X-100 and 0.1 % β-mercapto-ethanol). After centrifugation (20,000 *g*, 15 min, 4 °C) inclusion bodies were additionally washed in buffer W2 (20 mM Tris, pH 7.0, 0.5 % Triton X-100, 1 mM EDTA, 0.1 % β-mercapto-ethanol) and finally in buffer W3 (50 mM Tris, pH 7.0, 1 mM EDTA, 0.1 % β-mercapto-ethanol). Solubilization of the purified inclusion body proteins was achieved by resuspension in buffer medium S (10 mM Tris, pH 7.0, 0.1 mM EDTA, 1 mM DTT, 0.05 % polyethylene glycol 4000) containing 1.67 % of the detergent n-lauroylsarcosine and incubation for 15 min on ice. The protein fraction was diluted (threefold) with 10 mM Tris (pH 7.0) and finally, the solubilized proteins were separated from insoluble aggregates by centrifugation (12,000 g, 4 min, 4 °C).

### Preparation of proteoliposomes and transport measurements

For preparation of proteoliposomes 100 μg of the solubilized proteins were mixed with 20 mM Hepes, pH 7.0 and 1 mM PMSF. To obtain vesicles with internal counter exchange substrates 5 mM of phosphate or adenine nucleotides were added to the protein mixture. Preparation of mixed detergent-lipid micelles (100 mM PIPES, pH 7.0, 20 mg phosphatidylcholine, 1.6 mg cardiolipin, 28 mg C_10_E_5_) and detergent removal by amberlite XAD-2 beads was performed exactly as given by Heimpel *et al*., [34]. Overnight incubation with biobeads completed protein refolding and proteoliposome formation. External buffer medium and loading substrates were removed from the vesicles (500 μL) by desalting with NAP-5 columns (GE Healthcare; http://www.gehealthcare.com). Columns were equilibrated and liposomes were eluted with of import buffer (50 mM NaCl, 10 mM PIPES, pH 7.5). For transport measurements 50 μL of these proteoliposomes were mixed with 50 μL of import buffer supplemented with the indicated concentrations of [α^32^P]-ATP, [α^32^P]-ADP, [^45^Ca], MgCl_2_ and CaCl_2_ and incubated at 30 °C. At the given time points import was terminated by removal of external import medium via vacuum filtration as described in [35]. Briefly, liposomes were loaded to pre-wetted filters (mixed cellulose ester, 0.45-μm pore size; Whatman) and washed rapidly with phosphate buffer. Imported radioactivity was quantified by scintillation counting (Beckman LS6500; Beckman Coulter). For [^45^Ca] uptake measurements import was terminated and non-imported Ca^2+^ was removed by EGTA addition (2 mM) and incubation for 15 s prior to vacuum filtration and washing.

## Results

### Recombinant plant APCs act as ATP, ADP and P_i_ antiporters

To determine functional properties of the different APCs from *A. thaliana* we used the heterologous *Escherichia coli* expression system for production of the respective isoforms and performed transport measurements after carrier reconstitution into artificial lipid vesicles, so called liposomes. This approach was previously successfully applied to biochemically characterize several MCF proteins, including two selected human SCaMC isoforms [12, 34, 36–38].

The three plant APCs were heterologously expressed as N-terminal His-tag fusions. Like previously observed for many MCF proteins [12, 34, 36–38] also plant APCs were synthesized at high levels and accumulated in form of insoluble inclusion bodies (Additional file 1: Figure S1A and B). The aggregated proteins were enriched, purified, solubilized and finally refolded during their integration into liposomes.

**Fig. 1:**
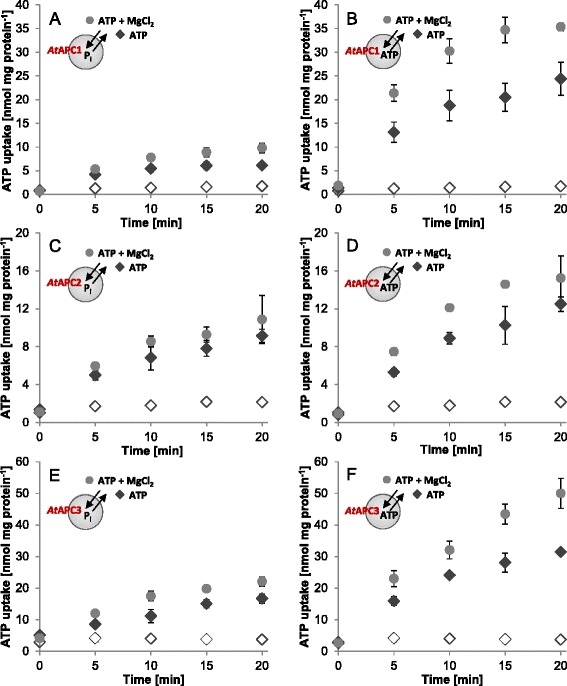
Time dependent ATP transport via *At*APC1-3. Transport of 50 μM [α^32^P]-ATP into P_i_ (**a**, **c**, **e**) and into ATP (**b**, **d**, **f**) loaded proteoliposomes with reconstituted *At*APC1 (**a**, **b**), *At*APC2 (**c**, **d**) and *At*APC3 (**e**, **f**). ATP uptake was measured in absence (black rhombs) and presence (gray circles) of 500 μM externally applied MgCl_2_. Non-loaded liposomes (non-filled rhombs; negative control) showed only marginal accumulation of radioactivity and the corresponding rates were unaffected by MgCl_2_ addition. Data represent mean values of at least three independent replicates, standard errors are given

Import measurements were performed on proteoliposomes either harboring or lacking selected possible counter exchange substrates in the lumen (Fig. 1, Additional file 2: Figure S2). This allowed investigation of *in vitro* transport activities and hence functionality of the reconstituted proteins as well as of the catalyzed transport mode. All recombinant plant APCs mediated time dependent uptake of [α^32^P]-ATP into phosphate (P_i_) loaded liposomes (Fig. 1a, c, e, black rhombs) and no comparable accumulation of radioactivity was observable with corresponding vesicles lacking P_i_ in the lumen (Fig. 1a, c, e, open rhombs). This observation already demonstrates that plant APCs can act as antiporters; ATP/P_i_ exchange by the different APC isoforms was linear for at least 5 min. Maximal uptake via *At*APC1 of ~ 6 nmol/mg protein was reached after 10 to 15 min (Fig. 1a, black rhombs), whereas *At*APC2 and *At*APC3 show marginally or considerably higher transport rates that approached a maximum of ~ 9 nmol/mg protein and ≥ 17 nmol/mg protein after 20 min, respectively (Fig. 1c and e, black rhombs).

Yeast Sal1p and mammalian SCaMCs were shown to discriminate against free ATP as substrate or at least to prefer the Mg^2+^-complexed form of ATP over free ATP [12, 15, 16, 39]. To check whether this is also true for the plant APCs, the influence of Mg^2+^ on ATP transport was analyzed. To this end, the ATP transport medium was supplemented with 500 μM Mg^2+^ to convert ~ 80 % of free ATP (ATP^4−^) into the Mg^2+^-complexed form (ATP-Mg^2−^) (http://maxchelator.stanford.edu/CaMgATPEGTA-TS.htm [40]). ATP-Mg^2−^ and HPO_4_^2−^ exchange results in an electroneutral transport. In case of *At*APC1 and *At*APC3 addition of Mg^2+^ caused moderate (~1.6-fold to 2.0-fold) increase in adenine nucleotide/P_i_ exchange compared to ATP without Mg^2+^ (Table 1; Fig. 1a and e, compare gray circles and black rhombs) whereas transport by *At*APC2 was stimulated to a lesser extent (Table 1; Fig. 1c, compare gray circles and black rhombs).

**Table 1:** Comparison of counter exchange rates of *At*APC1-3

Exchange (import/export)	*At*APC1	*At*APC2	*At*APC3
ATP/P_i_	4.1	5.0	7.2
ATP-Mg/P_i_	6.4	6.7	13.5
ATP/ATP	17.4	7.7	20.1
ATP-Mg/ATP	28.9	10.3	28.1
ADP/P_i_	7.6	5.7	11.3
ADP/ADP	39.8	12.2	42.0

ATP and ADP transport was allowed for 10 min. Rates represent net values of transport (minus corresponding transport into non-loaded liposomes) and are given in nmol/mg protein. For investigation of Mg^2+^ impact on ATP uptake 500 μM of MgCl_2_ were added to the transport medium. The complete time courses of ATP and ADP transport are displayed in Fig. 1 and in Additional file 2: Figure S2

To unravel whether the stimulatory influence of Mg^2+^ on ATP uptake is due to general preference for ATP-Mg as substrate or rather due to the electroneutrality of the corresponding transport process we investigated Mg^2+^-effects on ATP homo-exchange. Homo-exchange of ATP is electroneutral but becomes electrogenic when ATP-Mg^2−^ is exchanged with ATP^4−^. Comparison of the transport rates indicates that *At*APC1 highly, *At*APC3 markedly and *At*APC2 slightly prefer ATP homo-exchanges over the corresponding ATP/P_i_ hetero-exchanges (Table 1; compare Fig. 1a, c, e with b, d, f, black rhombs). Moreover, ATP homo-exchanges of all three *At*APCs became further enhanced by Mg^2+^ (Fig. 1b, d, f, compare gray circles and black rhombs) and the degree of Mg^2+^-dependent stimulation was nearly identical to that of the ATP/P_i_ hetero-exchange (Table 1). The observed stimulatory effects of Mg^2+^ on ATP/P_i_ and ATP/ATP transport indicate that *At*APC1 and 3 generally prefer ATP-Mg as substrate whereas *At*APC2 apparently only slightly favors the Mg^2+^-complexed form.

Because ADP represents an additional substrate of yeast Sal1p and human SCaMCs [12, 15, 18, 39] we verified whether this nucleotide is also transported by the plant orthologs in our *in vitro* system. For this, uptake of radiolabeled ADP into differentially loaded liposomes was measured. All plant APCs transported ADP in hetero-exchange with P_i_ as well as in homo-exchange with ADP and no import occurred into non-loaded vesicles (Additional file 2: Figure S2). The rates of ADP transport (in exchange with P_i_ or ADP) largely resemble the rates of the corresponding Mg^2+^-stimulated ATP transport (in exchange with P_i_ or ATP) (Table 1; compare Additional file 2: Figure S2 and Fig. 1). Just like observed for ATP transport, all plant APCs favor the homo-exchange of ADP over the corresponding ADP/P_i_ hetero-exchange and this preference is highly pronounced for *At*APC1 followed by *At*APC3 and finally *At*APC2 (Table 1). Moreover, comparison of the rates of ATP and ADP homo-exchanges with those of the corresponding P_i_ hetero-exchanges (Table 1) suggests that *At*APC2 in contrast to *At*APC1 and 3 does not strongly discriminate between nucleotides or P_i_ as internal counter exchange substrate. The ineffectiveness of non-loaded vesicles to induce significant import of ATP or ADP also demonstrates that vesicles do not allow carrier-independent passage of the labeled compounds.

### Calcium differentially affects ATP and ADP transport properties of the plant APCs

Diverse physiological data indicate a Ca^2+^-dependent regulation of mitochondrial net adenine nucleotide passage [16–18, 39, 41]. In the native environment many factors such as activity of adenylate kinases, Ca^2+^-induced metabolic processes, the mitochondrial membrane potential, respiration, Mg^2+^ complexation of ATP, etc. influence internal and external adenylate and P_i_ pools and consequently also mitochondrial adenine nucleotide translocation in general [42–45].

Transport studies with reconstituted APCs might provide a suitable tool to overcome interfering metabolic and physiological effects and to study the impact of Ca^2+^on this process in more detail. However, it is important to mention that transport of reconstituted human SCaMC1 was not stimulated by Ca^2+^ addition [12] and also *At*APC1-3 are already active in the absence of any Ca^2+^ addition (Fig. 1 and Additional file 2: Figure S2). These findings suggest that Ca^2+^ is not essentially required for carrier activation or that Ca^2+^contaminations exist in the buffer media. Determination of cations (by ion chromatography) revealed that in fact traces of both, Ca^2+^ and Mg^2+^, are present in the media (~9 μM, respectively).

If Ca^2+^ is essential for carrier activation and under the assumption that the proteoliposomes still contain a certain amount of inactive (Ca^2+^-free) APC proteins, an addition of extra Ca^2+^ should result in transport stimulation. To investigate a possible Ca^2+^-induced increase in transport activation we performed uptake studies with and without 200 μM Ca^2+^. Elevated Ca^2+^ availability generally stimulated nucleotide uptake of all three plant APCs (Table 2). This observation might point to a Ca^2+^-induced activation of previously inactive (Ca^2+^-free) carrier proteins. Studies with the two separately expressed sub-domains (N-terminal domain and membrane spanning part) of the human SCaMC1 led to the conclusion that the N-terminal domain acts as a lid that either opens or closes the translocation pathway in response to Ca^2+^ availability [22]. Given that Ca^2+^ exclusively causes removal of the N-terminal domain and hence activation of previously closed carriers, the same degree of stimulation would be expected independent of the kind of substrate exchanged. However, direct comparison of Ca^2+^ influence on different exchanges shows that for the reconstituted plant APCs the degree of stimulation is higher for ATP than for ADP or ATP-Mg uptake (Table 2).

**Table 2:** Stimulation of the given exchanges by addition of 200 μM CaCl_2_

Exchange (import/export)	*At*APC1	*At*APC2	*At*APC3
ATP/P_i_	3.05	3.12	4.30
ATP-Mg/P_i_	1.67	2.75	1.94
ATP/ATP	2.65	3.39	3.62
ATP-Mg/ATP	1.59	2.37	1.82
ADP/P_i_	1.55	2.03	1.49
ADP/ADP	1.56	2.12	1.54
ADP/ATP	1.79	2.20	1.70

Ca^2^^+^ −dependent stimulation (x-fold) was calculated according to corresponding transport in absence of Ca^2^^+^. SE are always below 15 % of the given value

We thus determined the apparent biochemical parameters of ATP/P_i_ and ADP/ATP exchange for all three *At*APCs in more detail (Table 3). Velocity of transport of all recombinant carriers approached saturation with increasing ATP or ADP concentrations and conformed to simple Michaelis-Menten kinetics (Additional file 3: Figure S3). The individual *At*APC isoforms differed in their respective ADP affinities (*At*APC1: 180 μM, *At*APC2: 374 μM and *At*APC3: 72 μM) whereas the ATP affinities were more similar (ranging from 68 to 113 μM). Affinities of *At*APC1 for ATP and ADP remained more or less unaffected by Ca^2+^ addition whereas ATP affinities of *At*APC2 and *At*APC3 increased (1.6- and 2.0-fold) and ADP affinities decreased (1.4- and 1.9-fold), respectively. All *At*APC isoforms generally exhibit lower maximal velocities (V_max_) for ATP than for ADP transport (Table 3). Since the V_max_ is proportional to the amount of actively transporting carrier proteins enhanced Ca^2+^-dependent activation of the APCs should be reflected by an identical increase in the V_max_ of both, ADP and ATP transport. However, addition of extra Ca^2+^ caused only a moderate increase (by approximately 1.2- to 1.5-fold) in maximal ADP V_max_ but stimulated the respective ATP V_max_ (2.0- to 2.5-fold) of all three APCs to a greater extend.

**Table 3:** Effects of 200 μM calcium on K_M_-Values of adenine nucleotide transport

Exchange	*At*APC1		*At*APC2		*At*APC3	
	K_M_	V_max_	K_M_	V_max_	K_M_	V_max_
ATP/P_i_	68 (6)	201(±14)	95 (±12)	212 (±24)	113 (±17)	282 (±20)
ATP/P_i_ + Ca^2+^	61 (±6)	398 (±30)	59 (±6)	523 (±48)	58 (±8)	646 (±88)
ADP/ATP	180 (±15)	2078 (±315)	374 (±40)	778 (±82)	72 (±5)	770 (±90)
ADP/ATP + Ca^2+^	178 (±15)	2455 (±357)	508 (±94)	1169 (±126)	140 (±12)	1025 (±140)

Transport was performed with rising ATP or ADP concentrations and allowed for 2.5 min. K_M_- values are given in μM and V_max_ in nmol mg protein^-1^ h^-1^. Data represent the mean of at least three independent experiments. Standard errors are given in brackets

The different effects of Ca^2+^ on ATP and ADP transport properties indicate that besides its proposed function in cap removal and carrier activation Ca^2+^ fulfills an additional role in substrate transport/recognition.

### Ca^2+^ effects override Mg^2+^ effects on ATP transport

To approach the function of Ca^2+^ during plant APC mediated transport it is important to keep in mind that ATP can form a complex with Mg^2+^as well as with Ca^2+^ and it is thus imaginable that plant APCs are capable of ATP-Ca transport *in vitro*.

**Fig. 2:**
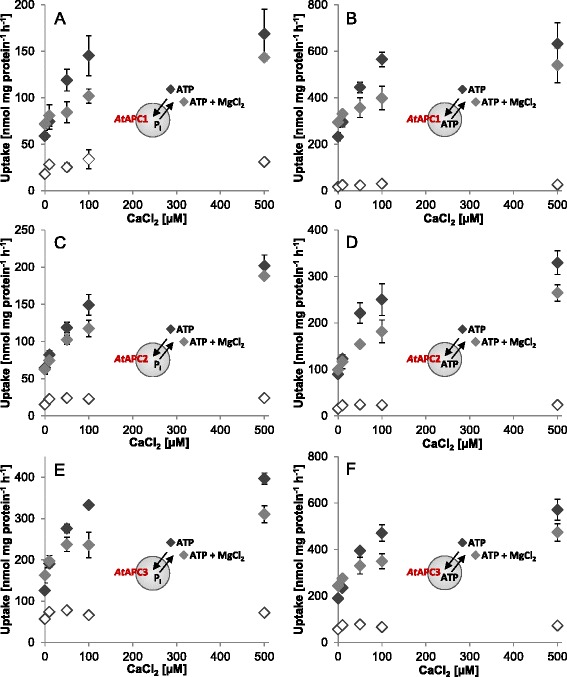
Ca^2+^-impact on ATP transport via *At*APC1-3. Effect of rising Ca^2+^-concentrations (0–500 μM) on transport mediated by recombinant *At*APC1 (**a**, **b**), *At*APC2 (**c**, **d**) and *At*APC3 (**e**, **f**). Transport of 50 μM [α^32^P]-ATP was conducted in absence (black rhombs) and presence of supplemental MgCl_2_ (gray rhombs). Transport was allowed for 5 min and is given in nmol mg protein^−1^ h^−1^. Ca^2+^-dependent stimulation of ATP/P_i_ hetero-exchanges (**a**, **c**, **e**) and ATP/ATP homo-exchanges (**b**, **d**, **f**). Non-loaded liposomes (non-filled rhombs; negative control) showed only marginal accumulation of ATP and the corresponding rates were unaffected by MgCl_2_ addition. Data represent mean values of three independent replicates, standard errors are given

Comparison of Ca^2+^ effects on ATP and ATP-Mg transport indeed revealed interesting results that support this assumption. Mg^2+^ addition causes marginal (*At*APC2) to moderate (*At*APC1 and 3) increase in ATP transport when no extra Ca^2+^ is present (Figs. 1 and 2). With rising Ca^2+^ concentration the positive impact of Mg^2+^ becomes abolished and even reverted into a negative one (Fig. 2). More precisely, with higher Ca^2+^ concentrations (>10 μM *At*APC2; > 50 μM *At*APC1 and 3) the rates of ATP transport in absence of Mg^2+^ exceed the rates of the corresponding exchange in presence of Mg^2+^. Accordingly, in presence of Mg^2+^ higher concentrations of Ca^2+^ are apparently required to achieve ATP-transport saturation.

### ATP transport stimulation by Ca^2+^ does not involve the N-terminal domain

We choose *At*APC2 for a more detailed analysis of the proposed ATP-Ca transport because ATP uptake of this transporter was markedly stimulated by Ca^2+^and particularly because Ca^2+^ stimulation was only slightly affected by Mg^2+^ presence (Fig. 2). To investigate ATP-Ca transport disconnected from possible Ca^2+^-dependent carrier activation we generated an *At*APC2 mutant protein lacking the predicted N-terminal domain (Additional file 4: Figure S4A and B). ATP uptake measurements verified that truncated *At*APC2 is functional (Additional file 4: Figure S4C), however, the uptake rates were slightly lower than those of the full-length protein.

**Fig. 3:**
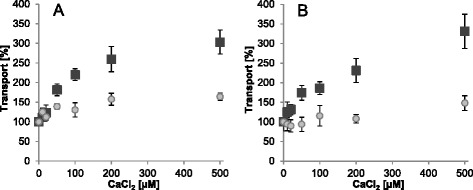
Ca^2+^-impact on ATP and ADP transport of full-length and N-terminally truncated *At*APC2. Transport via recombinant *At*APC2 (**a**) and via the mutated version lacking its N-terminal domain (**b**). Import of [α^32^P]-ATP into P_i_ loaded proteoliposomes (black squares) and of [α^32^P]-ADP into ATP loaded vesicles (gray circles) was allowed for 5 min. Transport without CaCl_2_ was set to 100 % and transport in presence of rising concentrations of externally added CaCl_2_ (0 - 500 μM) was calculated accordingly. Data represent net values of ATP/P_i_ and ADP/ATP uptake minus the respective control (non-loaded vesicles) of three independent replicates. Standard errors are given

Determination of Ca^2+^ impact on transport activity showed that ATP/P_i_ exchange via the mutated carrier was considerably stimulated by increasing Ca^2+^ concentrations (~3-fold). Moreover, the degree of Ca^2+^-dependent stimulation and the general course of the corresponding transport basically resembled that of the full-length protein (Fig. 3, black squares). Investigation of ADP uptake into ATP loaded liposomes revealed slight transport stimulation of the full-length protein by low Ca^2+^ concentrations (~35 % at 50 to 100 μM Ca^2+^), which approached saturation at higher concentrations (+60 %) (Fig. 3a, gray circles), whereas the corresponding transport of the truncated carrier version remained rather unaffected by moderate Ca^2+^ concentrations (+/− 10 % until 200 μM Ca^2+^) and became stimulated only at higher Ca^2+^ concentrations (+50 %) (Fig. 3b, gray circles).

Although slight differences in the Ca^2+^-impact are detectable, the higher influence of Ca^2+^ on ATP than on ADP import is apparently independent of the presence or absence of the N-terminal domain. This result verifies that the observed Ca^2+^-dependent ATP transport stimulation does not primarily result from carrier activation and might rather be caused by increased ATP-Ca formation and substrate availability.

### ATP but not ADP import of *At*APC2 requires the presence of divalent cations

Because full-length *At*APC2 already exhibits basic ATP/P_i_ exchange activity without extra Ca^2+^ addition and particularly because ADP uptake becomes not highly stimulated by rising Ca^2+^-concentrations (Fig. 3), it might be assumed that the majority of reconstituted carriers is already opened/activated due to contaminating Ca^2+^.

**Fig. 4:**
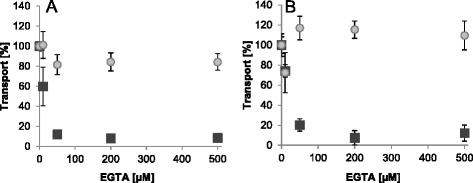
Impact of rising EGTA concentrations on adenine nucleotide transport of full-length and N-terminally truncated *At*APC2. Transport via recombinant *At*APC2 (**a**) and via the mutated version lacking its N-terminal domain (**b**). Import of [α^32^P]-ATP into P_i_ loaded proteoliposomes (black squares) and of [α^32^P]-ADP into ATP loaded vesicles (gray circles) was allowed for 5 min. Transport without EGTA was set to 100 % and transport in presence of rising concentrations of externally added EGTA (0 - 500 μM) was calculated accordingly. Data represent net values of ATP/P_i_ and ADP/ATP import minus the respective control (non-loaded vesicles) of three independent replicates. Standard errors are given

The cation chelator EGTA efficiently chelates Ca^2+^ (with significant higher affinity than to Mg^2+^) and accordingly should remove residual Ca^2+^ from the medium. We thus used addition of EGTA to the transport medium to investigate whether and how Ca^2+^ depletion affects carrier activities. ATP/P_i_ exchange of full-length *At*APC2 becomes significantly reduced by addition of 10 μM EGTA and further increase of its concentration causes total inhibition (Fig. 4a, black squares). Interestingly, a similar inhibitory effect was also observed for the truncated carrier version (Fig. 4b, black squares). Given that the N-terminal domain forms a lid that virtually closes the translocation pathway when free Ca^2+^ is missing, efficient Ca^2+^-removal should impede transport activity of *At*APC2 but not of the “un-capped” mutant. Moreover, ADP/ATP exchange of both, full-length and truncated, *At*APC2 variants remained more or less unaltered by EGTA addition (Fig. 4a and b, gray circles). Accordingly, Ca^2+^ removal from the medium did not cause inhibition of the overall transport capacity by deactivation of the reconstituted carrier.

**Fig. 5:**
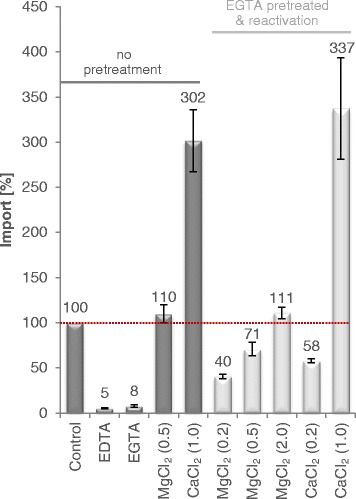
EGTA-inhibition of ATP transport via *At*APC2 and reactivation by MgCl_2_ and CaCl_2_. Transport of 50 μM [α^32^P]-ATP into P_i_ loaded vesicles in absence of EGTA, MgCl_2_ and CaCl_2_ was set to 100 % (control; red line). Transport in presence of 200 μM EGTA, 200 μM EDTA and the given concentrations (in mM) of MgCl_2_ or CaCl_2_ was calculated accordingly. Generally transport was allowed for 10 min. However, EGTA-inhibited transport was reactivated after 10 min of uptake by subsequent addition of MgCl_2_ or CaCl_2_ and transport was again allowed for 10 min (EGTA pretreatment and reactivation; light gray bars). Data represent net values (ATP import in exchange with P_i_ minus background values of non-loaded proteoliposomes) and are the mean of three independent experiments. Standard errors are indicated

Interestingly, transport via *At*APC2 was not only blocked by EGTA but also by the divalent cation chelator EDTA. Moreover, activity of the EGTA-inhibited carrier could be fully restored by either Ca^2+^ or Mg^2+^ (Fig. 5). However, when compared to Ca^2+^ higher concentrations of Mg^2+^ are required for transport reactivation/stimulation.

So far we cannot explain explicitly why solely ATP transport, but not general carrier activity, becomes inhibited by EGTA. It is imaginable that full-length *At*APC2 proteins are primarily or exclusively inserted in an inside-out orientation, exposing the N-terminal domain to the liposomal interior. This orientation would clearly hinder EGTA access to the regulatory sites (EF-Hands). However, *At*APC2-proteoliposomes loaded with P_i_ and 200 μM EGTA were still capable for ATP import (78 % of the corresponding EGTA-unaffected transport) (Additional file 5: Figure S5). Moreover, inhibition of ATP uptake into these EGTA-loaded liposomes by external EGTA as well as its (re)activation by 500 μM external Ca^2+^ were nearly identical when compared to standard *At*APC2-proteoliposomes lacking internal EGTA (Additional file 5: Figure S5).

Together, the obtained results indicate that ATP transport but not ADP or P_i_ transport of *At*APC2 essentially requires the presence of divalent cations and this requirement is independent of the N-terminal domain and thus not connected to carrier activation.

### Plant APC2 can mediate Ca^2+^-transport *in vitro*

The observed Ca^2+^ and EGTA effects on *At*APC2 activity led us to the conclusion that Ca^2+^ might act as an important co-substrate in ATP transport. To verify the proposed capacity of *At*APC2 for ATP-Ca transport in the liposomal system we performed uptake studies with 20 μM [^45^Ca] and 100 μM non-labeled ATP. Preliminary analyses revealed that the read-out of the import rates was hampered due to the high degree of nonspecific [^45^Ca]-interaction with the phospholipids at the liposomal surface (causing high radioactive background values). However, reduction of these non-specific background counts by removal of the vast majority of [^45^Ca] from the liposomal surface was achieved by additional EGTA treatment of the vesicles subsequent to the uptake measurements (prior to vacuum filtration and washing). The correspondingly modified transport assay allowed determination of small but significant time dependent Ca^2+^ uptake by full-length and truncated *At*APC2.

**Fig. 6:**
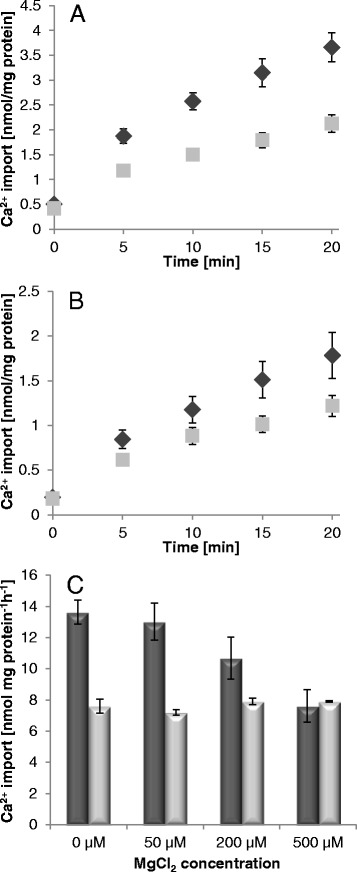
Determination of Ca^2+^ transport via *At*APC2. Time dependent uptake of [^45^Ca] via full-length *At*APC2 (**a**) and via N-terminally truncated *At*APC2 (**b**) reconstituted into P_i_ (black rhombs) and non-loaded liposomes (gray squares). (**c**) Effects of rising MgCl_2_ concentrations on [^45^Ca] transport into P_i_ loaded (dark gray bars) and non-loaded (light gray bars) *At*APC2 proteoliposomes. Transport media contained 20 μM [^45^Ca] and were additionally supplemented with 100 μM non-labeled ATP and the indicated concentrations of MgCl_2_. For determination of the Mg^2+^-effects on Ca^2+^ transport via *At*APC2 uptake was allowed for 10 min (given as nmol mg protein^−1^ h^−1^). Data represent mean values of three independent replicates. Standard errors are indicated

Ca^2+^ uptake into P_i_ loaded vesicles (Fig. 6a and b, black rhombs) always exceeded the corresponding rates obtained with non-loaded proteoliposomes (Fig. 6a and b, gray squares) indicating that Ca^2+^ accumulation is directly connected to the antiport activity of the carrier. The full-length protein exhibits higher Ca^2+^ transport rates and also the back-ground values of the non-loaded vesicles are enhanced when compared to the truncated version (compare Fig. 6a and b). So far it cannot be discriminated whether - albeit EGTA treatment - a certain amount of Ca^2+^ still binds to the N-terminal domain of recombinant *At*APC2 or/and the functionality of the truncated protein is generally slightly impaired.

Lastly, we analyzed effects of Mg^2+^ on Ca^2+^ import via recombinant *At*APC2. For this, P_i_ loaded and non-loaded *At*APC2 proteoliposomes were incubated in transport medium containing 20 μM [^45^Ca], 100 μM non-labeled ATP and increasing concentrations of Mg^2+^. [^45^Ca] import into phosphate loaded vesicles became significantly reduced by Mg^2+^ whereas the corresponding rates of the non-loaded vesicles remained more or less unaffected by Mg^2+^ addition (Fig. 6c). Quite high amounts of Mg^2+^ (200 μM) are required to cause approximately half maximal transport inhibition whereas 25-fold excess of Mg^2+^ completely blocks Ca^2+^ uptake. Because of the generally low [^45^Ca] transport rates of the truncated *At*APC2 reliable interpretation of the corresponding results obtained with this protein is complicated. Nevertheless, the tendency of Mg^2+^ impact on Ca^2+^ uptake generally resembles that of the full-length protein (Additional file 6: Figure S6). The obtained data suggest that Mg^2+^ competes with Ca^2+^ during ATP complex formation and thereby can reduce ATP-Ca availability and hence Ca^2+^-import via the reconstituted carrier.

## Discussion

### Transport capacities of plant APCs allow energy exchange as well as net adenine nucleotide provision

Diverse biological conditions, such as ATP-loading during mitochondrial biogenesis or physiological and environmental changes, require modulation of the mitochondrial adenine nucleotide pool size [9, 17, 18, 46]. During the past decades net influx or efflux of adenine nucleotides into or out of the organelle as well as the involved carriers have been well studied in mammals and yeast [9, 11, 12, 14–18, 46]. However, much less is known about these processes in plants.

It is quite obvious that also plant mitochondria have to adapt the adenine nucleotide concentration in the mitochondrial matrix in accordance to the respective metabolic demands. Already in the 1970s isolated corn and cauliflower mitochondria were shown to exhibit (carboxy)atractyloside insensitive (AAC independent) uptake of adenine nucleotides [26–28]. In the beginning, net import of ADP into plant mitochondria was identified to occur via exchange with P_i_ [31]. Later on, ADP transport was shown to be influenced by Mg^2+^ and Ca^2+^ and it was suggested that exogenous rather than endogenous P_i_ drives net ADP uptake [29]. These inconsistencies might be due to the fact that mitochondria harbor various carriers and enzymes directly or indirectly involved in adenine nucleotide transport and metabolism and that these proteins are differently affected by the respective test conditions and metabolic states of the organelle.

*Arabidopsis thaliana* encodes three MCF proteins (*At*APC1-3) that represent promising candidates for net adenine nucleotide transport. First of all, *At*APC1-3 exhibit significant amino acid similarities to APCs from animals or yeast and contain the characteristic N-terminal domain with EF-hand motifs (Additional file 7: Figure S7 and Additional file 8: Figure S8). Secondly, these proteins can compensate the growth defect of yeast ∆*sal1p* mutants inhibited in AAC mediated transport [32]. Thirdly, transport assays performed in this work with the reconstituted, recombinant carriers revealed that *At*APC1-3 act in a strict antiport mode (Fig. 1, Additional file 2: Figure S2); they can catalyze homo-exchanges of ATP and ADP as well as ATP/ADP hetero-exchange but most importantly also ATP and ADP hetero-exchange with P_i_*in vitro* (Fig. 1, Additional file 2: Figure S2, Tables 1, and 3). The latter capacity was also shown recently in a study by Palmieri and coworkers that was published while this manuscript was in revision [47]. Based on the *in vitro* characteristics growth-restoration in the yeast complementation assay by the three *At*APC isoforms [32] can be attributed to their capacity for net adenine nucleotide supply (complementation of Sal1p activity) and/or for energy provision (complementation of AAC activity).

Plant mitochondria possess a high affinity ADP uptake system that is sensitive to AAC-specific inhibitors and a low affinity ADP uptake system that apparently does not involve AAC activity [30]. Biochemical characterization of single isoforms suggest that AAC proteins mediate the high affinity ADP transport [48] whereas APCs catalyze or contribute to the low affinity ADP transport (Table 3) [47].

Interestingly, APC genes show more or less ubiquitous expression with highest rates in growing tissues of enhanced mitochondrial propagation (Aramemnon, BAR eFP browser; [49, 50]). The recent work by Palmieri and coworkers showed that the promoter of *Atapc1* exhibits enhanced activity when compared to the remaining two APC isoforms [47]. Moreover, expression of specific isoforms (Aramemnon, GENEVESTIGATOR [49, 51]) is induced by growth-promoting plant steroids (brassinosteroides) or in response to abiotic stressors, like hypoxia or phosphate limitation; conditions assumed to be associated with altered mitochondrial metabolism/respiration [45, 47, 52–54]. In future studies it will be interesting to determine whether specific developmental stages or stress situations characterized by enhanced or reduced APC expression correlate with the establishment or alteration of the mitochondrial adenine nucleotide pool.

### Substrate preferences and impact of divalent cations on transport

The fact that recombinant *At*APC3 and *At*APC1 apparently prefer homo-exchanges of ATP and ADP over the corresponding hetero-exchanges with P_i_ (Fig. 1, Additional file 2: Figure S2) might be indicative of transport reduction due to unfavorable charge imbalances generated in the liposomes by the electrogenic hetero-exchange. Similar to net ATP uptake by yeast and mammalian mitochondria [11, 15, 16, 18] ATP transport of *At*APC1 and *At*APC3 is markedly stimulated by Mg^2+^ (Fig. 1, Table 1). This stimulation occurs during homo- and hetero-exchange and suggests that *At*APC1 and *At*APC3 generally prefer ATP-Mg^2−^ over ATP^4−^ as import substrate independent of the generation of charge imbalances.

In contrast to *At*APC1 and *At*APC3, rates of homo- and hetero-exchange of recombinant *At*APC2 are quite similar (Fig. 1, Additional file 2: Figure S2) and ATP uptake was only slightly enhanced by Mg^2+^ addition (Fig. 1c and d). These observations suggest that either a strong preference of *At*APC2 for P_i_ as exchange substrate compensates possible negative effects of the charge imbalance of ATP/P_i_ (and ADP/P_i_) hetero-exchange or that hetero-exchange with P_i_ is not electrogenic at all. Interestingly, ATP transport of *At*APC2 was totally inhibited by EGTA or EDTA and could be restored by Mg^2+^ or Ca^2+^ (Fig. 5). This result strikingly argues for the requirement of divalent cations for ATP translocation. Whether this is due to their function as co-substrate and/or as effectors of the carrier protein cannot be unambiguously stated yet.

In contrast to our studies, Palmieri and coworkers investigated the capacity of ATP-Mg to act as export and not as import substrate and under those conditions ATP-Mg transport is rather unfavorable when compared to ATP [47]. Summarily, the current data therefore suggest that the plant APCs possess different substrate preferences at their exterior and interior side (Fig. 1, Table 1) [47].

**Fig. 7:**
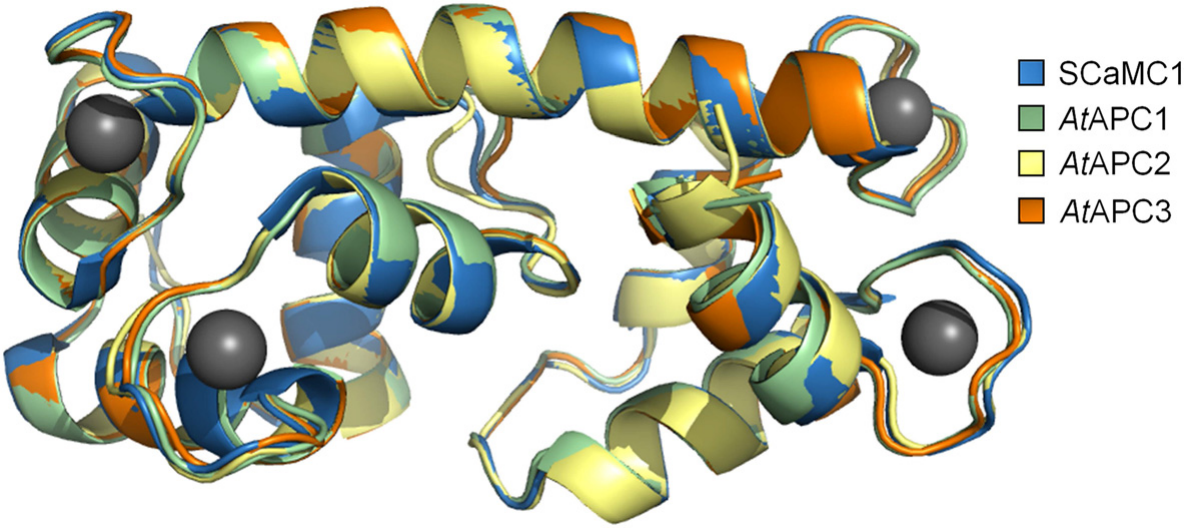
Structural alignment of the N-terminal domains of *At*APC1-3 and human SCaMC1. Three-dimensional homology models of *At*APC1 (residues 34–189, green), *At*APC2 (residues 38–194, yellow) and *At*APC3 (residues 35–189, orange). N-terminal domains were built using HHPred server and Modeller based on the crystal structure of the Ca^2+^-binding N-terminal domain of human SCaMC1 (blue; PDB ID: 4N5X) in complex with four calcium ions (gray spheres). The sequence alignment followed by a structural superimposition of the models was carried out using PyMOL (version 1.3)

Although Ca^2+^-dependent activity regulation of human and yeast APCs has been well known for a long time, first insights into the mechanistic principle were only gained recently. Sophisticated interaction studies with human SCaMC1 suggest that in absence of Ca^2+^ the quite flexible N-terminal domain caps the transmembrane part whereas Ca^2+^-binding turns the N-terminal domain into a more rigid state which leads to its dissociation and opening of the translocation pore [21, 22]. Superimposition of the corresponding regions in a structural alignment visualizes a high degree of conservation among the N-terminal domains of plant APCs and human SCaMC1 (Fig. 7). These structural similarities as well as computer based docking analyses (Additional file 8: Figure S8) suggest that the N-terminal domains of the plant APCs also interact with four Ca^2+^ ions. Moreover, amino acid sequence similarity to Sal1p and human SCaMC isoforms suggest that plant APCs are likewise regulated by Ca^2+^ (Additional file 7: Figure S7, Fig. 7).

The fact that reconstituted APC isoforms from human [12] and *A. thaliana* were already active without extra Ca^2+^-addition led to the assumption that Ca^2+^ contaminations in the buffer media were sufficient for carrier activation. Because increase in Ca^2+^-concentrations resulted in transport stimulation of all recombinant *At*APC isoforms one might conclude that under the reconstitution conditions a mix of active and non-active carries occurs and addition of Ca^2+^can thus activate additional carriers (Fig. 2). However, the rates of Ca^2+^-stimulation were not identical and varied depending on the kind of substrate transported (Table 2).

Assuming that Ca^2+^ exclusively operates in carrier activation by displacement of the N-terminal domain from the translocation pathway we would expect the same degree of (i) Ca^2+^-dependent transport stimulation, (ii) V_max_ increase (proportional to the amount of functional carriers), and (iii) transport reduction by Ca^2+^-depletion (with EGTA) independent of the exchanged substrates. Moreover, truncation of the N-terminal domain should cause constantly active carriers that are no longer influenced by Ca^2+^. However, the data obtained in this work suggest that this is not the case. We therefore hypothesize that in the *in vitro* system ATP-Ca acts as substrate of the plant APCs and is even favored over ATP-Mg or free ATP. By contrast, ADP-Ca seems to be rather discriminated against when compared to free ADP. Ca^2+^-induced alterations of the apparent transport affinities most likely reflect these specific substrate preferences of the respective APC isoforms e.g. higher preference for ATP-Ca (when compared with the Mg-complexed or free ATP) and lower preference of ADP-Ca (when compared to free ADP) (Table 3). Accordingly, Ca^2+^ complexation of ATP enhances and that of ADP reduces the amount of favored substrates and by this the respective transport capacity of the reconstituted protein. It is also imaginable that in the liposomal system, Ca^2+^ co-transport with ATP prevents charge accumulation of the ATP/P_i_ hetero-exchange and with ADP^3−^ (ADP-Ca^1−^) enhances the imbalance caused by the ADP/ATP hetero-exchange. In addition, effects of EGTA, EDTA, Mg^2+^ and Ca^2+^ on ATP transport inhibition, stimulation or reactivation suggest a competition between these cations during complex formation and provide further evidences for ATP-Ca as a potential *in vitro* substrate of recombinant plant APCs (Table 2 and Figs. 3, 4, 5). We conclude that the influence of Ca^2+^ on transport by the reconstituted APCs is a consequence of diverse factors, such as substrate preferences, charge accumulation/compensation and competition with Mg^2+^ during complex formation.

Transport characteristics obtained with *At*APC2 and the N-terminally truncated version support the assumption that ATP-transport stimulation by Ca^2+^ is not (or not exclusively) caused by activation of previously inactive (Ca^2+^-free) carriers. ATP transport of both, the full-length carrier and the truncated version, can be stimulated by Ca^2+^ and inhibited by EGTA whereas ADP transport was not significantly affected (Figs. 3 and 4). These results verify that solely ATP but not ADP transport activity is highly dependent on the presence of Ca^2+^ and that removal of this cation did not cause carrier deactivation in general. The ineffectiveness of EGTA in the inhibition of total transport activity is surprising. The possibility that plant APCs are generally not regulated in a Ca^2+^-dependent manner is apparently not applicable. Important structural similarities of the plant, yeast and mammalian isoforms are suggestive for a similar regulatory principle but most importantly, a corresponding regulation could be demonstrated in the recent study by Palmieri and coworkers [47]. It remains unclear whether in our *in vitro* system the functionality of the N-terminal domain of the recombinant *At*APC2 is somehow impaired or its affinity for Ca^2+^ is higher than that of EGTA. However, the possibility that insight-out orientation of reconstituted *At*APC2 and hence inaccessibility of the N-terminal domains caused ineffectiveness of EGTA in transport inhibition can be ruled out since proteoliposomes internally loaded with EGTA were still capable to import ATP in exchange with P_i_ (Additional file 5: Figure S5).

The fact that external but not internal EGTA caused inhibition of *At*APC2 mediated ATP import in exchange with P_i_ demonstrates that ATP but not P_i_ transport requires the presence of Ca^2+^ (or divalent cations). Moreover, this observation also demonstrates that the chelator at the liposomal interior is apparently physically separated from Ca^2+^ at the exterior (at least during the analyzed time span) which indicates that both, EGTA and Ca^2+^, do not pass the lipid barrier freely.

Notwithstanding or even because of the missing Ca^2+^-dependent regulation, we were able to identify the *in vitro* function of Ca^2+^ as co-substrate with the applied system.

Although uptake studies with α[^32^P]-ATP provided evidence for a possible ATP-Ca transport it would still have been imaginable that Ca^2+^ stimulates transport of unchelated ATP and impedes ATP-Mg transport in a different way. However, the specifically adapted uptake assay using [^45^Ca] provided a direct proof that ATP-Ca is *de facto* transported via reconstituted (Fig. 6). Time dependent uptake of [^45^Ca] via *At*APC2 is tightly connected to its antiport activity because P_i_ loaded proteoliposomes accumulated higher amounts of [^45^Ca] than non-loaded vesicles. Competition experiments further verified that ATP-Ca transport is favored over ATP-Mg transport *in vitro* since quite high concentrations of Mg^2+^ are required to reduce ATP-transport associated Ca^2+^ uptake (Fig. 6c, Additional file 6: Figure S6). When compared to full-length *At*APC2 the N-terminally truncated carrier shows reduced Ca^2+^ import capacity (Fig. 6b). Whether absence of the N-terminal domain affects transport activity directly or rather indirectly (via impairments in refolding and membrane insertion) cannot be deduced from these experiments.

Further studies with the reconstituted proteins as well as with transgenic APC plants and isolated mitochondria will be required to completely decipher, evaluate and compare *in vitro* and *in vivo* characteristics of APC proteins. Moreover, it will be interesting to determine the stoichiometry of the ATP and Ca^2+^ co-transport. Preliminary estimation suggests that these substrates are not transported in a 1:1 stoichiometry. However, in this context it is important to mention that uptake assays had to be adapted to make Ca^2+^ transport determination feasible and furthermore that Ca^2+^ and Mg^2+^ contaminations of the media have to be considered. Therefore, in future studies we want to further optimize Ca^2+^-transport measurements in liposomes and intent to decipher the impact of divalent cations on *At*APC1-3 function *in vivo*.

### Can ATP-Ca transport via plant APCs occur *in vivo*?

SCaMCs as well as yeast Sal1p seem to prefer ATP-Mg whereas our initial studies indicate that at least one of the *At*APC isoforms clearly favors ATP-Ca over both, ATP-Mg and ATP, as import substrate in the liposomal system. Due to the high structural similarity to ATP-Mg it is - from a biochemical point of view - not surprising that at least certain APCs can in principle accept ATP-Ca as substrate *in vitro*. However, the intriguing question arises whether ATP-Ca formation and correspondingly APC mediated Ca^2+^-transport can and will take place under physiological conditions. Generally, ATP-Ca formation is a rather unlikely phenomenon in plant cells. The concentration of free Ca^2+^ is usually low when compared to Mg^2+^, which represents a dominating divalent cation and also is Mg^2+^ favored over Ca^2+^ in ATP-complex formation. However, one could envision specific situations that might support possible ATP-Ca formation in close proximity to the carrier.

Although plant mitochondria contribute to Ca^2+^ storage, the majority of internal Ca^2+^ is probably transiently fixed as amorphous phosphate precipitate and thus the resting concentration of free Ca^2+^ in the matrix only slightly exceeds that of the cytosol (200 nM vs. 100 nM) [55–57]. Moreover, due to high Mg^2+^ concentrations within plant mitochondria ATP is nearly completely complexed with Mg^2+^, which argues against any potential ATP-Ca formation in the matrix [45]. Although lower Mg^2+^ levels in the cytosol increase the accessibility of free ATP, it is unclear whether conditions or microdomains of high Ca^2+^ availability at the mitochondrial surface might allow ATP-Ca formation [55, 58–63]. In the liposomal system Ca^2+^ uptake via *At*APC2 was low and completely blocked by 25-fold excess of Mg^2+^. If these characteristics (the biochemical properties in combination with a high Mg^2+^ to Ca^2+^ ratio next to the carrier) also represent the *in vivo* situation, ATP-Ca transport via plant APCs is highly unlikely to occur.

Although, a direct role of plant APCs in ATP-Ca transport is therefore arguable, recent data suggest an indirect function of a mammalian isoform in Ca^2+^ translocation. SCaMC3 was shown to physically interact with the (low affinity) Mitochondrial Calcium Uniporter (MCU) and lack of SCaMC3 apparently decreases ATP and Ca^2+^ import into mitochondria [24, 64]. Accordingly, SCaMC3 was supposed to represent an important component of the mitochondrial Ca^2+^ uptake system, a supercomplex formed by channels and carriers in microdomains for enhanced Ca^2+^-sensitivity [64]. Whether certain plant APC isoforms fulfill a function related to that described for SCaMC3 is unclear, however, physical proximity to proteins involved in Ca^2+^ release might be advantageous to guarantee fast Ca^2+^-dependent activation and response of plant APCs.

## Conclusions

Determination of the biochemical characteristics of three putative APC isoforms from *A. thaliana* in the liposomal system revealed that the recombinant carriers mediate ATP, ADP and phosphate exchange. Accordingly, plant mitochondria harbor a subset of carriers capable of net adenine nucleotide translocation, however in contrast to yeast and mammalian orthologs they show no high preference for ATP-Mg as import substrate. Surprisingly, we instead obtained evidence for a possible ATP-Ca transport by the reconstituted plant APCs in the liposomal context but it is arguable that physiological Mg^2+^ and Ca^2+^ concentrations most likely prevent ATP-Ca formation and its subsequent transport *in vivo*. Although we were not able to detect EF-hand based Ca^2+^-dependent carrier regulation, this was shown recently to exist in plant APCs [47]. Summarily, the current data suggest that low Ca^2+^ concentrations regulate activity of plant APCs via EF-hands of the N-terminal domain whereas high Ca^2+^ concentrations can induce its own transport as co-substrate of ATP *in vitro*. While this study deepens our knowledge about mitochondrial net nucleotide transport of plants it also gives rise to new intriguing questions. In the future, it is important to investigate the *in vivo* function of plant APCs and the impact of divalent cations on the corresponding transport.

## Additional files

Additional file 1: Figure S1. Heterologously expressed *At*APC1-3 proteins accumulate in the inclusion body fraction of *E. coli *expression cells. (**A**) SDS- PAGE of 5 μg and (**B**) Western-blot and immunodetection of 0.5 μg of the inclusion bodies fraction from cells expressing *At*APC1 (lanes 1), *At*APC2 (lanes 2) and *At*APC3 (lanes 3). The Western-blot was immuno-decorated with a monoclonal anti poly His IgG (Sigma, Taufkirchen, Germany). M, prestained molecular weight marker (Thermo Fisher Scientific, Schwerte, Germany) for estimation of the molecular masses (given in kDa) of the recombinant proteins. (PDF 66 kb) Additional file 2: Figure S2. Time dependent ADP transport via *At*APC1-3. Transport of 50 μM [α^32^P]-ADP into P_i_ (**A**, **C**, **E**) and into ADP (**B**, **D**, **F**) loaded proteoliposomes with reconstituted *At*APC1 (**A**, **B**), *At*APC2 (**C**, **D**) and *At*APC3 (**E**, **F**). Non-loaded liposomes (non-filled rhombs; negative control) showed only marginal accumulation of radioactivity when compared to proteoliposomes loaded with P_i_ or ADP (black rhombs). Data represent mean values of three independent replicates, standard errors are given. (PDF 82 kb) Additional file 3: Figure S3.
**a**. Determination of biochemical parameters of ATP import into P_i_ loaded APC-proteoliposomes. Transport of *At*APC1 (**A**, **B**), *At*APC2 (**C**, **D**) and *At*APC3 (**E**, **F**) was performed with rising ATP concentrations in absence (**A**, **C**, **E**) or presence (**B**, **D**, **F**) of 200 μM CaCl_2_ and allowed for 2.5 min. Michaelis-Menten kinetics are the mean of at least 3 replicates, SE are given. **b**. Determination of biochemical parameters of ADP import into ATP loaded APC-proteoliposomes. Transport of *At*APC1 (**A**, **B**), *At*APC2 (**C**, **D**) and *At*APC3 (**E**, **F**) was performed with rising ADP concentrations in absence (**A**, **C**, **E**) or presence (**B**, **D**, **F**) of 200 μM CaCl_2_ and allowed for 2.5 min. Michaelis-Menten kinetics are the mean of at least 3 replicates, SE are given. (PDF 126 kb) Additional file 4: Figure S4. Heterologous expression and ATP transport analysis of N- terminally truncated *At*APC2. (**A**) SDS-PAGE of 5μg and (**B**) Western-blot and immunodetection of 0.5 μg of the inclusion bodies fraction from *E. coli *cells expressing the N-terminally truncated (lanes 1). To enable detection of the molecular mass reduction due to loss of the N-terminal extension the full-length protein was included in this analysis (lanes 2). The Western-blot was immuno-decorated with a monoclonal anti poly His IgG (Sigma, Taufkirchen, Germany). M, prestained molecular weight marker (Thermo Fisher Scientific). (**C**) Time dependent import of 50 μM [α^32^P]-ATP via N- terminally truncated *At*APC2 into ATP loaded (black rhombs), P_i_ loaded (gray circles) and non-loaded (non-filled rhombs) liposomes. (PDF 156 kb) Additional file 5: Figure S5. Impact of internal EGTA on ATP transport via *At*APC2. Uptake of 50 μM [α^32^P]-ATP into proteoliposomes loaded with P_i_ (black bars) or P_i_ plus 200 μM EGTA (light gray bars) was set to 100% (control). Inhibitory and stimulatory effects of externally added EGTA (50 μM) and CaCl_2_ (500 μM) on the corresponding transport rates were calculated accordingly. Reactivation of transport inhibited by external EGTA was induced by addition of 500 μM CaCl_2_. Transport (inhibition as well as activation) was allowed for 10 min. Data represent net values (ATP/P_i_ exchange minus background values of non-loaded proteoliposomes) and are the mean of at least three replicates. Standard errors are indicated. (PDF 252 kb) Additional file 6: Figure S6. Effects of rising MgCl_2_ concentrations on [^45^Ca] transport via the N- terminally truncated *At*APC2. Transport of 20 μM [^45^Ca] into P_i_ loaded (dark gray bars) and non- loaded (light gray bars) proteoliposomes was allowed for 10 min (given as nmol mg protein^-1^ h^-^^1^). The transport medium was supplemented with 100 μM non-labeled ATP and the indicated MgCl_2_ concentrations. Data represent mean values of three independent replicates. Standard errors are indicated. (PDF 102 kb) Additional file 7: Figure S7. Alignment of APC proteins from different organisms. Amino acid sequence alignment of APCs from *A. thaliana* (*At*APC1-3 [GenBank:At5g61810; At5g51050; At5g07320]), *S. cerevisiae* (Sal1p [GenBank: YNL083w]) and human (*Hs*SCaMC1-3 [GenBank:SLC25A24; SLC25A25; SLC25A23] using ClustalW2 (http://www.ebi.ac.uk). To allow easy detection of the N-terminal extension mitochondrial AAC2 from *S. cerevisiae* (*Sc*PET9 [GenBank:YBL030C]) was included as a representative MCF protein. Shading of conserved amino acid residues was performed with Boxshade at the Swiss EMBnet server (http://www.ch.embnet.org/index.html). Residues of the N-terminal domains of *At*APC1-3 proposed to be involved in Ca^2^^+^-interaction are highlighted by different colors. Residues predicted by Scanprosite (http://prosite.expasy.org/scanprosite) are marked in green and by molecular Ca^2+^ docking analyses with AutoDock vina (see also Additional file 8: Figure S8) are marked in orange. Ca^2^^+^-interacting residues predicted by Scanprosite and molecular docking studies are marked in yellow. EF-hands I and III (orange boxes) exhibit lower support for Ca^2^^+^-interaction (Scanprosite) than EF-hands II and IV (green boxes). (PDF 476 kb) Additional file 8: Figure S8. Docking poses of Ca^2+^ ions within the N-terminal domains of *At*APC1-3, interacting residues and structural superimposition with human SCaMC1 (SLC25A24). Three-dimensional homology models of the N-terminal domains of *At*APC1 (residues 34-189, green), *At*APC2 (residues 38-194, yellow) and *At*APC3 (residues 35-189, orange) were built using HHPred server and Modeller using the crystal structure of the Ca^2^^+^-bound state of the N-terminal domain of human SCaMC1 (blue) as template (PDB ID: 4N5X). The four EF-hand motifs putatively involved in Ca^2^^+^ binding are marked in dark blue (**A**, **C**, **E**). Docking poses of Ca^2+^ ions are shown for *At*APC1 N-term (**A**), *At*APC2 N-term (**C**) and *At*APC3 N-term (**E**) with residues putatively interacting with Ca^2+^ marked in red. These residues were chosen either based on docking or Scanprosite results (http://prosite.expasy.org/scanprosite). For the molecular docking analyses, Ca^2+^ ions and the N-terminal domains of *At*APC1-3 were prepared using Autodock Tools 1.5.6. After determination of the search space, the ions were docked into the structures using Autodock vina. The best binding poses for Ca^2+^ were selected with respect to the total energy and EF-hand positions. Structural superimposition of *At*APC1 (**B**), *At*APC2 (**D**) and *At*APC3 (**F**) with SCaMC1 (blue) and Ca^2+^ ions within this protein (blue spheres) was carried out using PyMOL (version 1.3). (PDF 298 kb)
