# Supplementary material for: In vitro analyses of mitochondrial ATP/phosphate carriers from Arabidopsis thaliana revealed unexpected Ca2+-effects
Source: BMC Plant Biol. 2015 Oct 6;15:238. doi: 10.1186/s12870-015-0616-0 (PMC4595200; doi:10.1186/s12870-015-0616-0)
Supplement: Additional file 1: Figure S1. — Heterologously expressed AtAPC1-3 proteins accumulate in the inclusion body fraction of E. coli expression cells. (A) SDS- PAGE of 5 μg and (B) Western-blot and immunodetection of 0.5 μg of the inclusion bodies fraction from cells expressing AtAPC1 (lanes 1), AtAPC2 (lanes 2) and AtAPC3 (lanes 3). The Western-blot was immuno-decorated with a monoclonal anti poly His IgG (Sigma, Taufkirchen, Germany). M, prestained molecular weight marker (Thermo Fisher Scientific, Schwerte, Germany) for estimation of the molecular masses (given in kDa) of the recombinant proteins. (PDF 66 kb) [file 12870_2015_616_MOESM1_ESM.pdf]

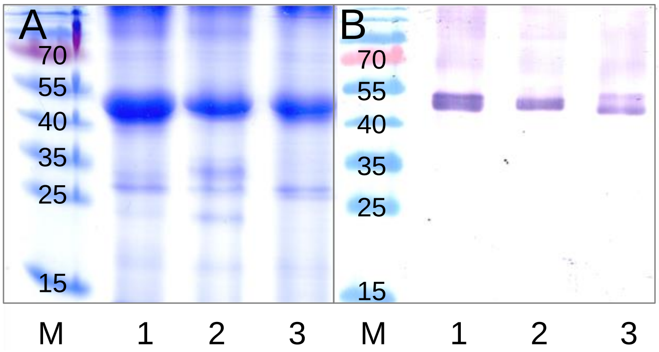

Supplementary Figure 1. Heterologously expressed *At*APC1-3 proteins accumulate in the inclusion body fraction of *E. coli* expression cells. (**A**) SDS-PAGE of 5 µg and (**B**) Western-blot and immunodetection of 0.5 µg of the inclusion bodies fraction from cells expressing *At*APC1 (lanes 1), *At*APC2 (lanes 2) and *At*APC3 (lanes 3). The Western-blot was immuno-decorated with a monoclonal anti poly His IgG (Sigma, Taufkirchen, Germany). M, prestained molecular weight marker (Thermo Fisher Scientific, Schwerte, Germany) for estimation of the molecular masses (given in kDa) of the recombinant proteins.
