# Supplementary material for: In vitro analyses of mitochondrial ATP/phosphate carriers from Arabidopsis thaliana revealed unexpected Ca2+-effects
Source: BMC Plant Biol. 2015 Oct 6;15:238. doi: 10.1186/s12870-015-0616-0 (PMC4595200; doi:10.1186/s12870-015-0616-0)
Supplement: Additional file 2: Figure S2. — Time dependent ADP transport via AtAPC1-3. Transport of 50 μM [α32P]-ADP into Pi (A, C, E) and into ADP (B, D, F) loaded proteoliposomes with reconstituted AtAPC1 (A, B), AtAPC2 (C, D) and AtAPC3 (E, F). Non-loaded liposomes (non-filled rhombs; negative control) showed only marginal accumulation of radioactivity when compared to proteoliposomes loaded with Pi or ADP (black rhombs). Data represent mean values of three independent replicates, standard errors are given. (PDF 82 kb) [file 12870_2015_616_MOESM2_ESM.pdf]

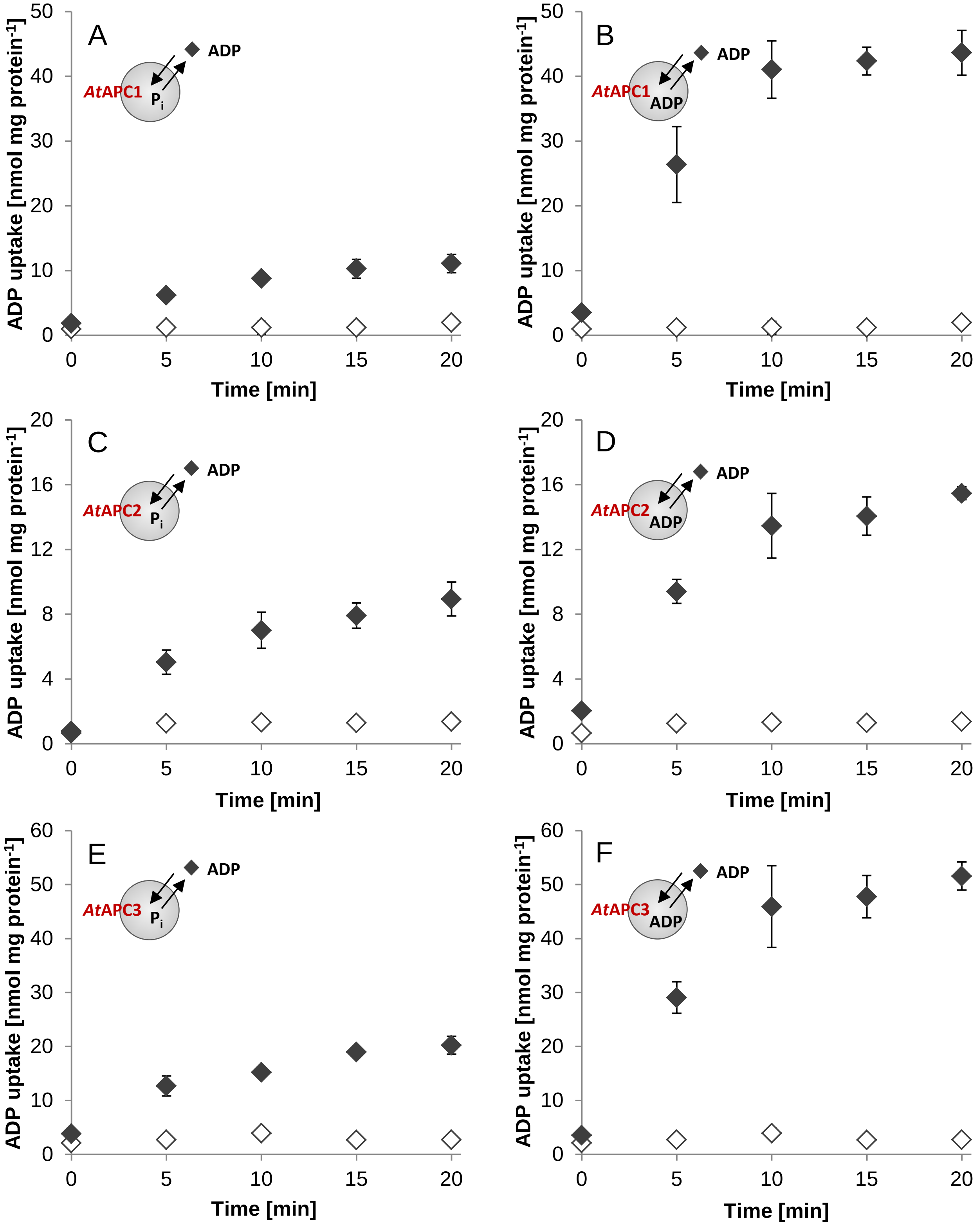

Supplementary Figure 2. Time dependent ADP transport via *At*APC1-3. Transport of 50 µM [α^{32}P]-ADP into P_i (**A, C, E**) and into ADP (**B, D, F**) loaded proteoliposomes with reconstituted *At*APC1 (**A, B**), *At*APC2 (**C, D**) and *At*APC3 (**E**, **F**). Non-loaded liposomes (non-filled rhombs; negative control) showed only marginal accumulation of radioactivity when compared to proteoliposomes loaded with P_i or ADP (black rhombs). Data represent mean values of three independent replicates, standard errors are given.
