# Supplementary material for: In vitro analyses of mitochondrial ATP/phosphate carriers from Arabidopsis thaliana revealed unexpected Ca2+-effects
Source: BMC Plant Biol. 2015 Oct 6;15:238. doi: 10.1186/s12870-015-0616-0 (PMC4595200; doi:10.1186/s12870-015-0616-0)
Supplement: Additional file 3: Figure S3. — a. Determination of biochemical parameters of ATP import into Pi loaded APC-proteoliposomes. Transport of AtAPC1 (A, B), AtAPC2 (C, D) and AtAPC3 (E, F) was performed with rising ATP concentrations in absence (A, C, E) or presence (B, D, F) of 200 μM CaCl2 and allowed for 2.5 min. Michaelis-Menten kinetics are the mean of at least 3 replicates, SE are given. b. Determination of biochemical parameters of ADP import into ATP loaded APC-proteoliposomes. Transport of AtAPC1 (A, B), AtAPC2 (C, D) and AtAPC3 (E, F) was performed with rising ADP concentrations in absence (A, C, E) or presence (B, D, F) of 200 μM CaCl2 and allowed for 2.5 min. Michaelis-Menten kinetics are the mean of at least 3 replicates, SE are given. (PDF 126 kb) [file 12870_2015_616_MOESM3_ESM.pdf]

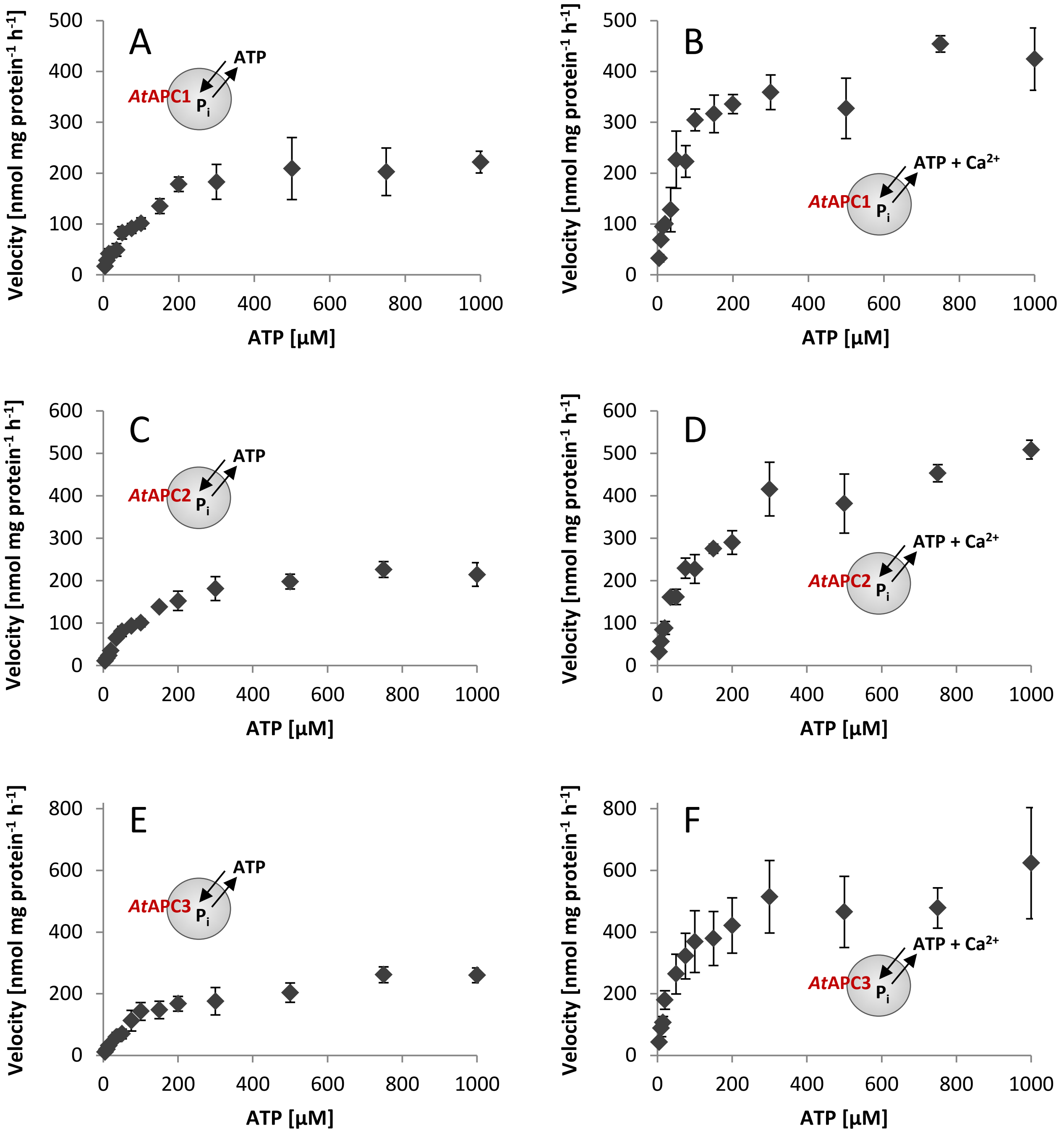

Supplementary Figure 3a. Determination of biochemical parameters of ATP import into P_i loaded APC-proteoliposomes. Transport of *At*APC1 (A, B), *At*APC2 (C, D) and *At*APC3 (E, F) was performed with rising ATP concentrations in absence (A, C, E) or presence (B, D, F) of 200 µM $CaCl_2$ and allowed for 2.5 min. Micheales-Menten kinetics are the mean of at least 3 replicates, SE are given.

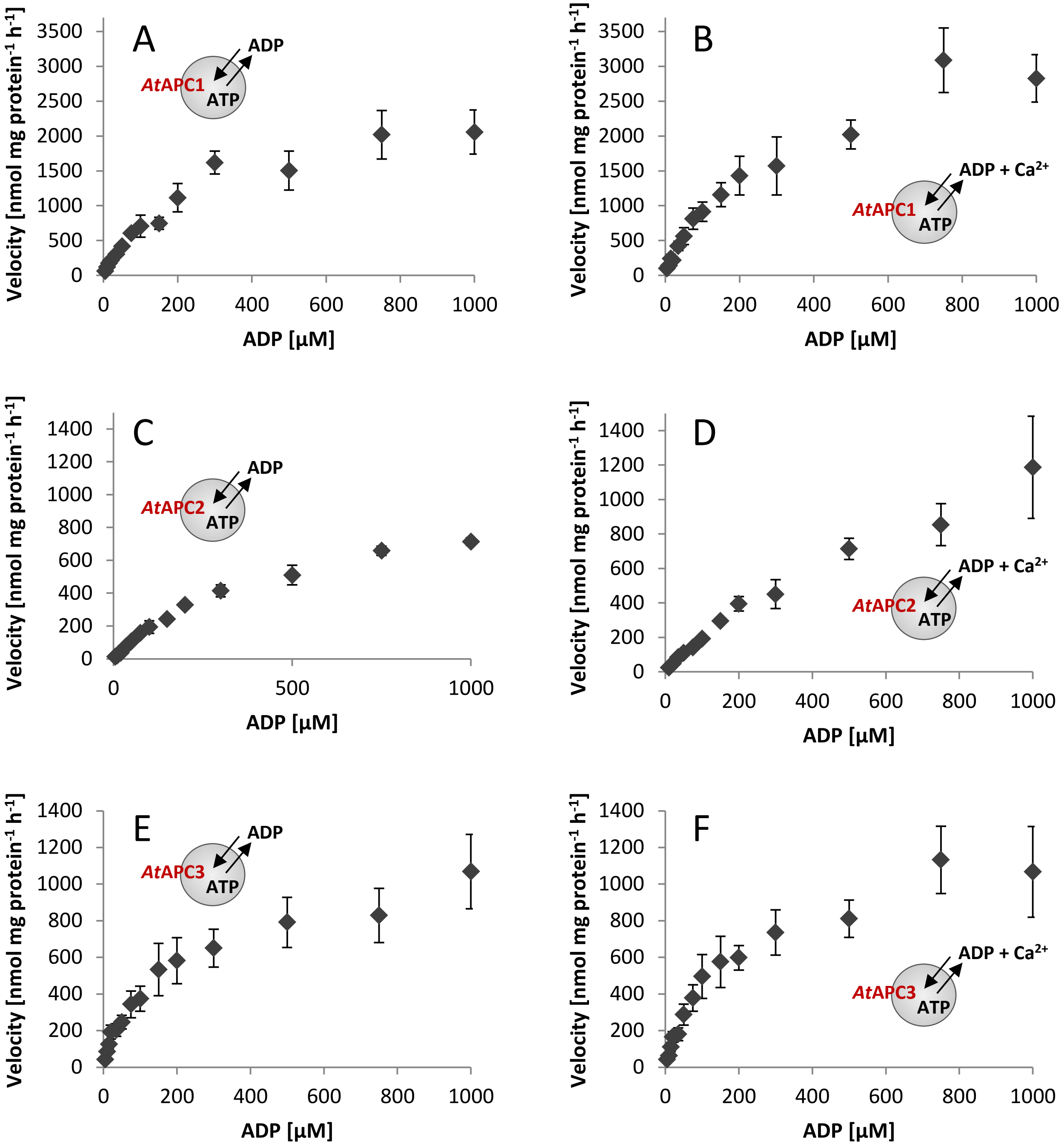

Supplementary Figure 3b. Determination of biochemical parameters of ADP import into ATP loaded APC-proteoliposomes. Transport of *At*APC1 (A, B), *At*APC2 (C, D) and *At*APC3 (E, F) was performed with rising ADP concentrations in absence (A, C, E) or presence (B, D, F) of 200 µM $CaCl_2$ and allowed for 2.5 min. Micheales-Menten kinetics are the mean of at least 3 replicates, SE are given.
