# Supplementary material for: In vitro analyses of mitochondrial ATP/phosphate carriers from Arabidopsis thaliana revealed unexpected Ca2+-effects
Source: BMC Plant Biol. 2015 Oct 6;15:238. doi: 10.1186/s12870-015-0616-0 (PMC4595200; doi:10.1186/s12870-015-0616-0)
Supplement: Additional file 4: Figure S4. — Heterologous expression and ATP transport analysis of N- terminally truncated AtAPC2. (A) SDS-PAGE of 5μg and (B) Western-blot and immunodetection of 0.5 μg of the inclusion bodies fraction from E. coli cells expressing the N-terminally truncated (lanes 1). To enable detection of the molecular mass reduction due to loss of the N-terminal extension the full-length protein was included in this analysis (lanes 2). The Western-blot was immuno-decorated with a monoclonal anti poly His IgG (Sigma, Taufkirchen, Germany). M, prestained molecular weight marker (Thermo Fisher Scientific). (C) Time dependent import of 50 μM [α32P]-ATP via N- terminally truncated AtAPC2 into ATP loaded (black rhombs), Pi loaded (gray circles) and non-loaded (non-filled rhombs) liposomes. (PDF 156 kb) [file 12870_2015_616_MOESM4_ESM.pdf]

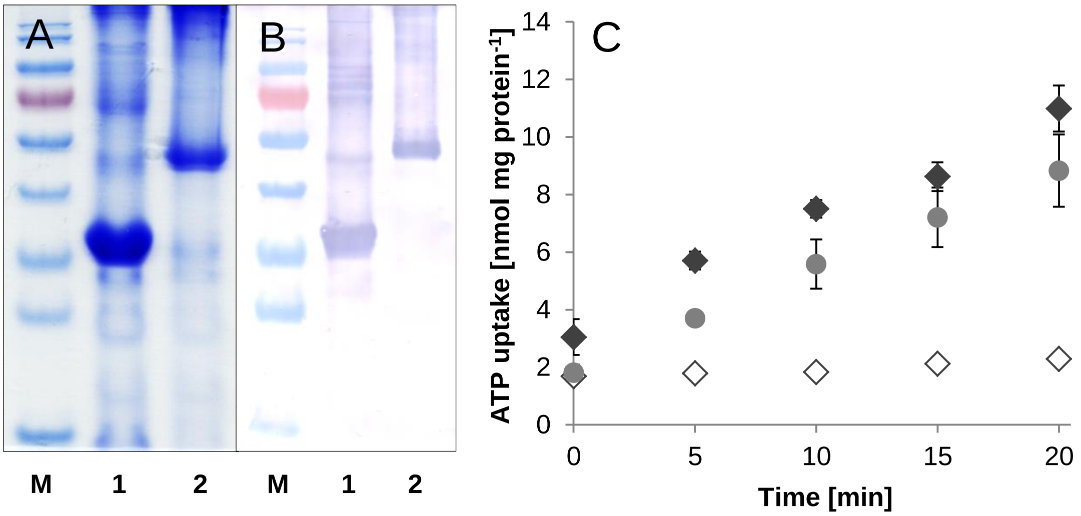

Supplementary Figure 4. Heterologous expression and ATP transport analysis of N-terminally truncated *At*APC2. (**A**) SDS-PAGE of 5µg and (**B**) Western-blot and immunodetection of 0.5 µg of the inclusion bodies fraction from *E. coli* cells expressing the N-terminally truncated *At*APC2 (lanes 1). To enable detection of the molecular mass reduction due to loss of the N-terminal extension the full-length protein was included in this analysis (lanes 2). The Western-blot was immuno-decorated with a monoclonal anti poly His IgG (Sigma, Taufkirchen, Germany). M, prestained molecular weight marker (Thermo Fisher Scientific). (**C**) Time dependent import of 50 µM [α^{32}P]-ATP via N-terminally truncated *At*APC2 into ATP loaded (black rhombs), P_i loaded (gray circles) and non-loaded (non-filled rhombs) liposomes.
