# Supplementary material for: In vitro analyses of mitochondrial ATP/phosphate carriers from Arabidopsis thaliana revealed unexpected Ca2+-effects
Source: BMC Plant Biol. 2015 Oct 6;15:238. doi: 10.1186/s12870-015-0616-0 (PMC4595200; doi:10.1186/s12870-015-0616-0)
Supplement: Additional file 5: Figure S5. — Impact of internal EGTA on ATP transport via AtAPC2. Uptake of 50 μM [α32P]-ATP into proteoliposomes loaded with Pi (black bars) or Pi plus 200 μM EGTA (light gray bars) was set to 100% (control). Inhibitory and stimulatory effects of externally added EGTA (50 μM) and CaCl2 (500 μM) on the corresponding transport rates were calculated accordingly. Reactivation of transport inhibited by external EGTA was induced by addition of 500 μM CaCl2. Transport (inhibition as well as activation) was allowed for 10 min. Data represent net values (ATP/Pi exchange minus background values of non-loaded proteoliposomes) and are the mean of at least three replicates. Standard errors are indicated. (PDF 252 kb) [file 12870_2015_616_MOESM5_ESM.pdf]

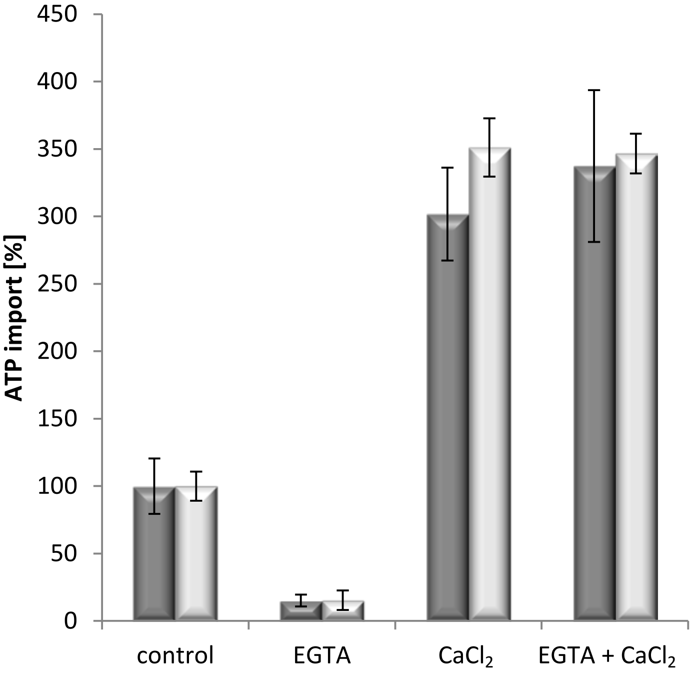

Supplementary Figure 5. Impact of internal EGTA on ATP transport via *At*APC2. Uptake of 50 µM [α^{32}P]-ATP into proteoliposomes loaded with P_i (black bars) or P_i plus 200 µM EGTA (light gray bars) was set to 100% (control). Inhibitory and stimulatory effects of externally added EGTA (50 µM) and $CaCl_2$ (500 µM) on the corresponding transport rates were calculated accordingly. Reactivation of transport inhibited by external EGTA was induced by addition of 500 µM $CaCl_2$. Transport (inhibition as well as activation) was allowed for 10 min. Data represent net values (ATP/P_i exchange minus background values of non-loaded proteoliposomes) and are the mean of at least three replicates. Standard errors are indicated.
