# Supplementary material for: In vitro analyses of mitochondrial ATP/phosphate carriers from Arabidopsis thaliana revealed unexpected Ca2+-effects
Source: BMC Plant Biol. 2015 Oct 6;15:238. doi: 10.1186/s12870-015-0616-0 (PMC4595200; doi:10.1186/s12870-015-0616-0)
Supplement: Additional file 6: Figure S6. — Effects of rising MgCl2 concentrations on [45Ca] transport via the N- terminally truncated AtAPC2. Transport of 20 μM [45Ca] into Pi loaded (dark gray bars) and non- loaded (light gray bars) proteoliposomes was allowed for 10 min (given as nmol mg protein-1 h- 1). The transport medium was supplemented with 100 μM non-labeled ATP and the indicated MgCl2 concentrations. Data represent mean values of three independent replicates. Standard errors are indicated. (PDF 102 kb) [file 12870_2015_616_MOESM6_ESM.pdf]

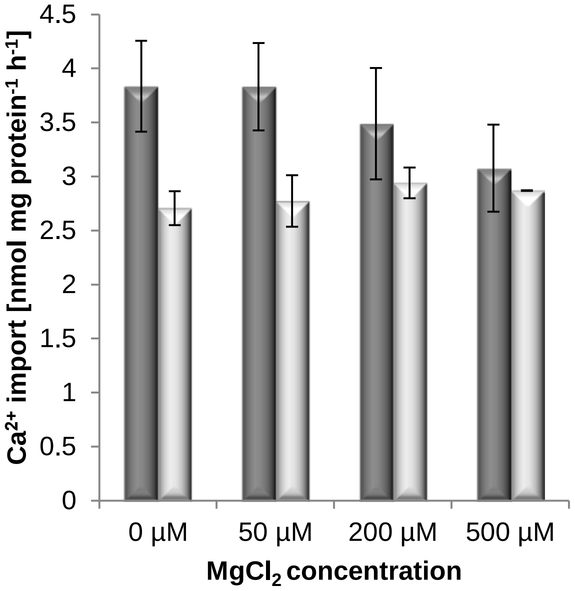

Supplementary Figure 6. Effects of rising $MgCl_2$ concentrations on [^{45}Ca] transport via the N-terminally truncated *At*APC2. Transport of 20 µM [^{45}Ca] into P_i loaded (dark gray bars) and non-loaded (light gray bars) proteoliposomes was allowed for 10 min (given as nmol mg protein^{-1} h^{-1}). The transport medium was supplemented with 100 µM non-labeled ATP and the indicated $MgCl_2$ concentrations. Data represent mean values of three independent replicates. Standard errors are indicated.
