# Supplementary material for: In vitro analyses of mitochondrial ATP/phosphate carriers from Arabidopsis thaliana revealed unexpected Ca2+-effects
Source: BMC Plant Biol. 2015 Oct 6;15:238. doi: 10.1186/s12870-015-0616-0 (PMC4595200; doi:10.1186/s12870-015-0616-0)
Supplement: Additional file 7: Figure S7. — Alignment of APC proteins from different organisms. Amino acid sequence alignment of APCs from A. thaliana (AtAPC1-3 [GenBank:At5g61810; At5g51050; At5g07320]), S. cerevisiae (Sal1p [GenBank: YNL083w]) and human (HsSCaMC1-3 [GenBank:SLC25A24; SLC25A25; SLC25A23] using ClustalW2 (http://www.ebi.ac.uk). To allow easy detection of the N-terminal extension mitochondrial AAC2 from S. cerevisiae (ScPET9 [GenBank:YBL030C]) was included as a representative MCF protein. Shading of conserved amino acid residues was performed with Boxshade at the Swiss EMBnet server (http://www.ch.embnet.org/index.html). Residues of the N-terminal domains of AtAPC1-3 proposed to be involved in Ca2 +-interaction are highlighted by different colors. Residues predicted by Scanprosite (http://prosite.expasy.org/scanprosite) are marked in green and by molecular Ca2+ docking analyses with AutoDock vina (see also Additional file 8: Figure S8) are marked in orange. Ca2 +-interacting residues predicted by Scanprosite and molecular docking studies are marked in yellow. EF-hands I and III (orange boxes) exhibit lower support for Ca2 +-interaction (Scanprosite) than EF-hands II and IV (green boxes). (PDF 476 kb) [file 12870_2015_616_MOESM7_ESM.pdf]

```
AtAPC1   : MEAEKSK----QNPGKKPVEATMEHVLVALRETKEKREIRIQKLFEFFDNSKLGFLDDTQIEKGLSSLSIP---PKYRYASDFLKVCD :  81
AtAPC2   : MEATKSSKQNCCNPVKKPGPVSIDHVLLALRETREERDLRIRSLFSFFDSENVGYLDCAQIEKGLCALQIP---SGYKYAKELFRVCD :  85
AtAPC3   : MESSKPKN---RNPMKKPVSITMEHVLLALRETMDEREIRIRSLFDFFDNSNLGFLDYAQIEKGLASLQIP---PEYKYARDLFRVCD :  82
HsSCAMC1 : -----------------MLRWLRDFVLPTAACQDAEQPTRYETLFQALDRNGDGVVDIGELQEGLRNLGIP---LGQDAEEKIFTTGD :  68
HsSCAMC2 : -----------------ML----------CLCLYVPVIGEAQTEFQYFES-KGLPAELKSIFKLSVFIPSQEFSTYRQWKQKIVQAGD :  60
HsSCAMC3 : -----------------MR----------GSPGDAERRQRWGRLFEELDSNKDGRVDVHELRQGLARLGGG--NPDPGAQQGISSEGD :  59
ScSAL1P  : -------------------------MLLKNCETDKQRDIRYACLFKELDVKGNGQVTLDNLISAFEKNDHP-LKGNDEAIKMLFTAMD :  62
ScPET9   : ------------------------------------------------------------------------------------------ :   -
                                                         EF-hand I                         EF-

AtAPC1   : SNRDGRVDYQEFRRYMDAKELELYKIFQAIDIEHNGDICPAELWEALDKAG------------------------------------IK : 134
AtAPC2   : ANRDGRVDYHEFRRYMDDKELELYRIFQAIDVEHNGCISPEGLWDSLVKAG------------------------------------IE : 138
AtAPC3   : ANRDGRVDYQEFRRYIDAKELELYRIFQAIDVEHNGCILPEELWEALVKAG------------------------------------IE : 135
HsSCAMC1 : VNKDGKLDFEEFMKYLKDHEKKMKLAFKSLDKNNDGKIEASEIVQSLQTLG------------------------------------LT : 121
HsSCAMC2 : KDLDGQLDFEEFVHYLQDHEKKLRLVFKSLDKKNDGRIDAQEIMQSLRDLG------------------------------------VK : 113
HsSCAMC3 : ADPDGGLDLEEFSRYLQEREQRLLLMFHSLDRNQDGHIDVSEIQQSFRALG------------------------------------IS : 112
ScSAL1P  : VNKDSVVDLSDFKKYASNAESQIWNGFQRIDLDHDGKIGINEINRYLSDLDNQSICNNELNHELSNEKMNKFSRFFEWAFPKRKANIA : 150
ScPET9   : ------------------------------------------------------------------------------------------ :   -
             hand II                    EF-hand III

AtAPC1   : IKDEELASFMEHVDK-----DNNGIITFEEWRDFLLLNPHEATIENIYHHWERVCLIDIGEQAVIPDGISAHAQRS----KLLLAGGI : 213
AtAPC2   : IKDEELARFVEHVDK-----DNDGIIMFEEWRDFLLLYPHEATIENIYHHWERVCLVDIGEQAVIPEGISKHIKRS----NYFIAGGI : 217
AtAPC3   : IDDEELARFVEHVDK-----DNNGTITFEEWRDFLLLYPHEATLENIYHHWERVCLIDIGEQAVIPDGISKHVKRS----RLLLAGGL : 214
HsSCAMC1 : ISEQQAELILQSIDV-----DGTMTVDWNEWRDYFLFNPVTD-IEEIIRFWKHSTGIDIGDSLTIPDEFTEDEKKSGQWWRQLLAGGI : 203
HsSCAMC2 : ISEQQAEKILKSMDK-----NGTMTIDWNEWRDYHLLHPVEN-IPEIILYWKHSTIFDVGENLTVPDEFTVEERQTGMWWRHLVAGGG : 195
HsSCAMC3 : ISLEQAEKILHSMDR-----DGTMTIDWQEWRDHFLLHSLEN-VEDVLYFWKHSTVLDIGECLTVPDEFSKQEKLTGMWWKQLVAGAV : 194
ScSAL1P  : LRGQASHKKNTDNDRSKKTTDSDLYVTYDQWRDFLLLVPRKQ-GSRLHTAYSYFYLFNEDVDLSSEGDVTLINDFIRG-FGFFIAGGI : 236
ScPET9   : -----------------------------------------------------MSSNAQVKTPLPPAPAPKKESNFLIDFLMGGV :  32
                       EF-hand IV

AtAPC1   : AGAVSRTATAPLDRLKVALQVQRTNLG------------------------VVPTIKKIWREDKLLGFFRGNGLNVAKVAPESAIKFA : 277
AtAPC2   : AGAASRTATAPLDRLKVLLQIQKTDAR------------------------IREAIKLIWKQGGVRGFFRGNGLNIVKVAPESAIKFY : 281
AtAPC3   : AGAVSRTATAPLDRLKVVLQVQRAHAG------------------------VLPTIKKIWREDKLMGFFRGNGLNVMKVAPESAIKFC : 278
HsSCAMC1 : AGAVSRTSTAPLDRLKIMMQVHGSKSDKMN---------------------IFGGFRQMVKEGGIRSLWRGNGTNVIKIAPETAVKFW : 270
HsSCAMC2 : AGAVSRTCTAPLDRLKVLMQVHASRSNNMG---------------------IVGGFTQMIREGGARSLWRGNGINVLKIAPESAIKFM : 262
HsSCAMC3 : AGAVSRTGTAPLDRLKVFMQVHASKTNRLN---------------------ILGGLRSMVLEGGIRSLWRGNGINVLKIAPESAIKFM : 261
ScSAL1P  : SGVISRTCTAPFDRLKVFLIARTDLSSILLNSKTDLLAKNPNADINKISSPLAKAVKSLYRQGGIKAFYVGNGLNVIKVFPESSIKFG : 324
ScPET9   : SAAVAKTAASPIERVKLLIQNQDEMLKQGTLDRKYAG--------------ILDCFKRTATQEGVISFWRGNTANVIRYFPTQALNFA : 106

AtAPC1   : AYEMLKPIIG----GAD-GDIGTSGRLLAGGLAGAVAQTAIYPMDLVKTRLQTFVSEVG------TPKLWKLTKDIWIQEGPRAFYRG : 354
AtAPC2   : AYELFKNAIGENM-GEDKADIGTTVRLFAGGMAGAVAQASIYPLDLVKTRLQTYTSQAGVA----VPRLGTLTKDILVHEGPRAFYKG : 364
AtAPC3   : AYEMLKPMIG----GED-GDIGTSGRLMAGGMAGALAQTAIYPMDLVKTRLQTCVSEGGK-----APKLWKLTKDIWVREGPRAFYKG : 356
HsSCAMC1 : AYEQYKKLLT-----EEGQKIGTFERFISGSMAGATAQTFIYPMEVMKTRLAVGKTGQYS-------GIYDCAKKILKHEGLGAFYKG : 346
HsSCAMC2 : AYEQIKRLVG-----SDQETLRIHERLVAGSLAGAIAQSSIYPMEVLKTRMALRKTGQYS-------GMLDCARRILAREGVAAFYKG : 338
HsSCAMC3 : AYEQIKRAIL-----GQQETLHVQERFVAGSLAGATAQTIIYPMEVLKTRLTLRRTGQYK-------GLLDCARRILEREGPRAFYRG : 337
ScSAL1P  : SFEVTKKIMTKLEGCRDTKDLSKFSTYIAGGLAGMAAQFSVYPIDTLKFRVQCAPLDTKLK---GNNLLFQTAKDMFREGGLRLFYRG : 409
ScPET9   : FKDKIKAMFGFKK--EEGYAKWFAGNLASGGAAGALSLLFVYSLDYARTRLAADSKSSKKGGARQFNGLIDVYKKTLKSDGVAGLYRG : 192

AtAPC1   : LCPSLIGIIPYAGIDLAAYETLKDLSRA------HFLHDTAEPGPLIQLGCGMTSGALGASCVYPLQVIRTRMQADSSK------TSM : 430
AtAPC2   : LFPSLLGIIPYAGIDLAAYETLKDLSRT------YILQDAE-PGPLVQLGCGTISGALGATCVYPLQVVRTRMQAERAR------TSM : 439
AtAPC3   : LFPSLLGIVPYAGIDLAAYETLKDLSRT------YILQDTE-PGPLIQLSCGMTSGALGASCVYPLQVVRTRMQADSSK------TTM : 431
HsSCAMC1 : YVPNLLGIIPYAGIDLAVYELLKSYWLD------NFAKDSVNPGVMVLLGCGALSSTCGQLASYPLALVRTRMQAQAMLEG-SPQLNM : 427
HsSCAMC2 : YVPNMLGIIPYAGIDLAVYETLKNAWLQ------HYAVNSADPGVFVLLACGTMSSTCGQLASYPLALVRTRMQAQASIEG-APEVTM : 419
HsSCAMC3 : YLPNVLGIIPYAGIDLAVYETLKNWWLQ------QYSHDSADPGILVLLACGTISSTCGQIASYPLALVRTRMQAQASIEG-GPQLSM : 418
ScSAL1P  : VTVGIVGIFPYAALDLGTFSALKKWYIAKQAKTLNLPQDQVTLSNLVVLPMGAFSGTVGASVVYPINLLRTRLQAQGTYAHPYVYNGF : 497
ScPET9   : FLPSVVGIVVYRGLYFGMYDSLKPLLLT----------GSLEGSFLASFLLGWVVTTGASTCSYPLDTVRRRMMMTSGQAV--KYDGA : 268

AtAPC1   : GQEFLKTLRGEGLKGFYRGIFPNFFKVIPSASISYLVYEAMKKNLALD-- : 478
AtAPC2   : SGVFRRTISEEGYRALYKGLLPNLLKVVPAASITYMVYEAMKKSLELD-- : 487
AtAPC3   : KQEFMNTMKGEGLRGFYRGLLPNLLKVVPAASITYIVYEAMKKNMALD-- : 479
HsSCAMC1 : VGLFRRIISKEGIPGLYRGITPNFMKVLPAVGISYVVYENMKQTLGVTQK : 477
HsSCAMC2 : SSLFKHILRTEGAFGLYRGLAPNFMKVIPAVSISYVVYENLKITLGVQSR : 469
HsSCAMC3 : LGLLRHILSQEGMRGLYRGIAPNFMKVIPAVSISYVVYENMKQALGVTSR : 468
ScSAL1P  : KDVLLKTLEREGYQGLFKGLVPTLAKVCPAVSISYLCYENLKKFMNLE-- : 545
ScPET9   : FDCLRKIVAAEGVGSLFKGCGANILRGVAGAGVISMYDQLQMILFGKKFK : 318
```

**Supplementary Figure 7.** Alignment of APC proteins from different organisms. Amino acid sequence alignment of APCs from *A. thaliana* (*At*APC1-3 [GenBank:At5g61810; At5g51050; At5g07320]), *S. cerevisiae* (Sal1p [GenBank: YNL083w]) and human (*Hs*SCaMC1-3 [GenBank:SLC25A24; SLC25A25; SLC25A23] using ClustalW2 (www.ebi.ac.uk). To allow easy detection of the N-terminal extension mitochondrial AAC2 from *S. cerevisiae* (*Sc*PET9 [GenBank:YBL030C]) was included as a representative MCF protein. Shading of conserved amino acid residues was performed with Boxshade at the Swiss EMBnet server (http://www.ch.embnet.org/index.html). Residues of the N-terminal domains of *At*APC1-3 proposed to be involved in $Ca^{2+}$-interaction are highlighted by different colors. Residues predicted by Scanprosite (http://prosite.expasy.org/scanprosite) are marked in green and by molecular $Ca^{2+}$ docking analyses with AutoDock vina (see also Supplementary Figure 8) are marked in orange. $Ca^{2+}$-interacting residues predicted by Scanprosite and molecular docking studies are marked in yellow. EF-hands I and III (orange boxes) exhibit lower support for $Ca^{2+}$-interaction (Scanprosite) than EF-hands II and IV (green boxes).
