# Supplementary material for: In vitro analyses of mitochondrial ATP/phosphate carriers from Arabidopsis thaliana revealed unexpected Ca2+-effects
Source: BMC Plant Biol. 2015 Oct 6;15:238. doi: 10.1186/s12870-015-0616-0 (PMC4595200; doi:10.1186/s12870-015-0616-0)
Supplement: Additional file 8: Figure S8. — Docking poses of Ca2+ ions within the N-terminal domains of AtAPC1-3, interacting residues and structural superimposition with human SCaMC1 (SLC25A24). Three-dimensional homology models of the N-terminal domains of AtAPC1 (residues 34-189, green), AtAPC2 (residues 38-194, yellow) and AtAPC3 (residues 35-189, orange) were built using HHPred server and Modeller using the crystal structure of the Ca2 +-bound state of the N-terminal domain of human SCaMC1 (blue) as template (PDB ID: 4N5X). The four EF-hand motifs putatively involved in Ca2 + binding are marked in dark blue (A, C, E). Docking poses of Ca2+ ions are shown for AtAPC1 N-term (A), AtAPC2 N-term (C) and AtAPC3 N-term (E) with residues putatively interacting with Ca2+ marked in red. These residues were chosen either based on docking or Scanprosite results (http://prosite.expasy.org/scanprosite). For the molecular docking analyses, Ca2+ ions and the N-terminal domains of AtAPC1-3 were prepared using Autodock Tools 1.5.6. After determination of the search space, the ions were docked into the structures using Autodock vina. The best binding poses for Ca2+ were selected with respect to the total energy and EF-hand positions. Structural superimposition of AtAPC1 (B), AtAPC2 (D) and AtAPC3 (F) with SCaMC1 (blue) and Ca2+ ions within this protein (blue spheres) was carried out using PyMOL (version 1.3). (PDF 298 kb) [file 12870_2015_616_MOESM8_ESM.pdf]

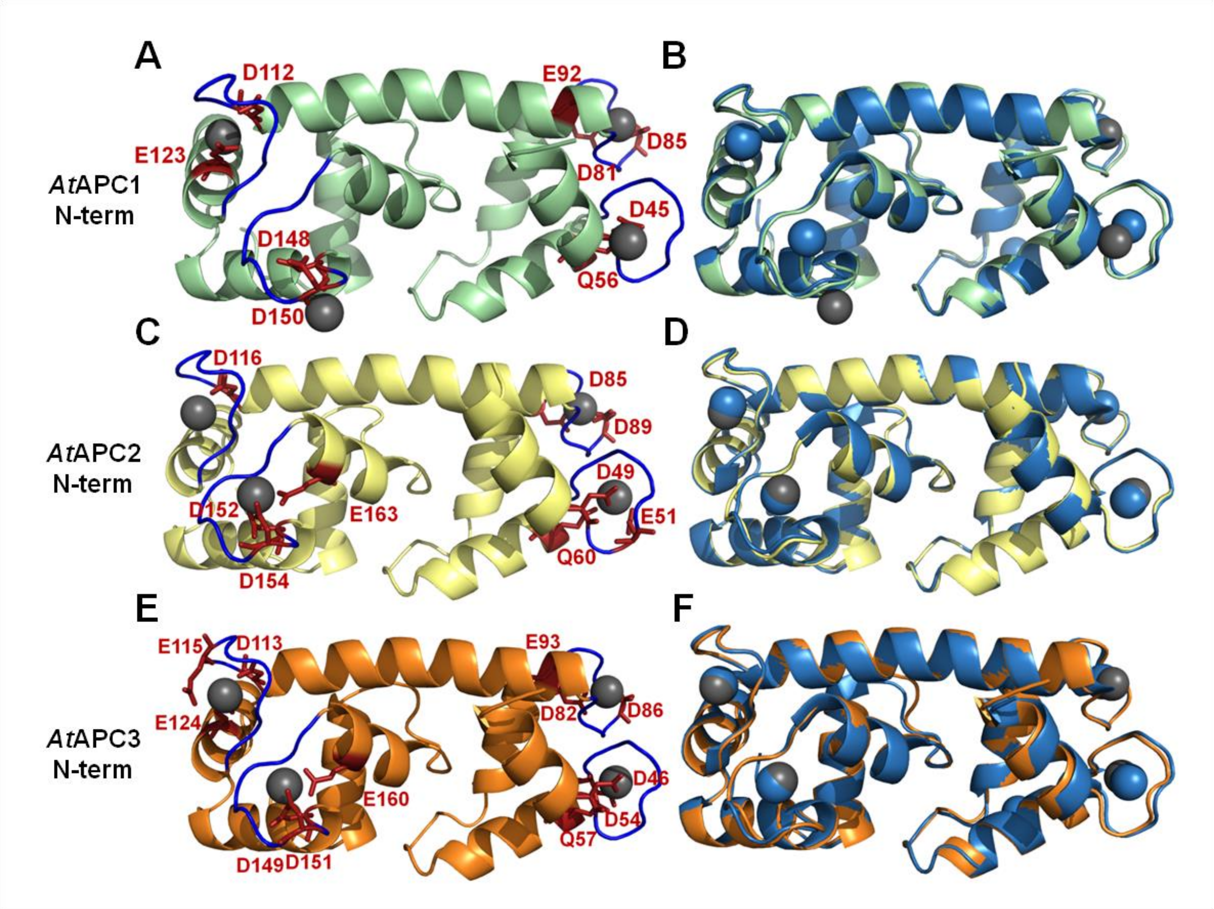

Supplementary Figure 8. Docking poses of Ca^{2+} ions within the N-terminal domains of *At*APC1-3, interacting residues and structural superimposition with human SCaMC1 (SLC25A24). Three-dimensional homology models of the N-terminal domains of *At*APC1 (residues 34-189, green), *At*APC2 (residues 38-194, yellow) and *At*APC3 (residues 35-189, orange) were built using HHPred server and Modeller using the crystal structure of the Ca^{2+}-bound state of the N-terminal domain of human SCaMC1 (blue) as template (PDB ID: 4N5X). The four EF-hand motifs putatively involved in Ca^{2+} binding are marked in dark blue (**A**, **C**, **E**). Docking poses of Ca^{2+} ions are shown for *At*APC1 N-term (**A**), *At*APC2 N-term (**C**) and *At*APC3 N-term (**E**) with residues putatively interacting with Ca^{2+} marked in red. These residues were chosen either based on docking or Scanprosite results (http://prosite.expasy.org/scanprosite). For the molecular docking analyses, Ca^{2+} ions and the N-terminal domains of *At*APC1-3 were prepared using Autodock Tools 1.5.6. After determination of the search space, the ions were docked into the structures using Autodock vina. The best binding poses for Ca^{2+} were selected with respect to the total energy and EF-hand positions. Structural superimposition of *At*APC1 (**B**), *At*APC2 (**D**) and *At*APC3 (**F**) with SCaMC1 (blue) and Ca^{2+} ions within this protein (blue spheres) was carried out using PyMOL (version 1.3).
